# Coxsackievirus B Exits the Host Cell in Shed Microvesicles Displaying Autophagosomal Markers

**DOI:** 10.1371/journal.ppat.1004045

**Published:** 2014-04-10

**Authors:** Scott M. Robinson, Ginger Tsueng, Jon Sin, Vrushali Mangale, Shahad Rahawi, Laura L. McIntyre, Wesley Williams, Nelson Kha, Casey Cruz, Bryan M. Hancock, David P. Nguyen, M. Richard Sayen, Brett J. Hilton, Kelly S. Doran, Anca M. Segall, Roland Wolkowicz, Christopher T. Cornell, J. Lindsay Whitton, Roberta A. Gottlieb, Ralph Feuer

**Affiliations:** 1 The Integrated Regenerative Research Institute (IRRI) at San Diego State University, Cell & Molecular Biology Joint Doctoral Program, Department of Biology, San Diego State University, San Diego, California, United States of America; 2 Donald P. Shiley BioScience Center, San Diego State University, San Diego, California, United States of America; 3 Department of Biology and Center for Microbial Sciences, San Diego State University, San Diego, California, United States of America; 4 Department of Immunology and Microbial Science, SP30-2110, The Scripps Research Institute, La Jolla, California, United States of America; National Institutes of Health, United States of America

## Abstract

Coxsackievirus B3 (CVB3), a member of the picornavirus family and enterovirus genus, causes viral myocarditis, aseptic meningitis, and pancreatitis in humans. We genetically engineered a unique molecular marker, “fluorescent timer” protein, within our infectious CVB3 clone and isolated a high-titer recombinant viral stock (Timer-CVB3) following transfection in HeLa cells. “Fluorescent timer” protein undergoes slow conversion of fluorescence from green to red over time, and Timer-CVB3 can be utilized to track virus infection and dissemination in real time. Upon infection with Timer-CVB3, HeLa cells, neural progenitor and stem cells (NPSCs), and C2C12 myoblast cells slowly changed fluorescence from green to red over 72 hours as determined by fluorescence microscopy or flow cytometric analysis. The conversion of “fluorescent timer” protein in HeLa cells infected with Timer-CVB3 could be interrupted by fixation, suggesting that the fluorophore was stabilized by formaldehyde cross-linking reactions. Induction of a type I interferon response or ribavirin treatment reduced the progression of cell-to-cell virus spread in HeLa cells or NPSCs infected with Timer-CVB3. Time lapse photography of partially differentiated NPSCs infected with Timer-CVB3 revealed substantial intracellular membrane remodeling and the assembly of discrete virus replication organelles which changed fluorescence color in an asynchronous fashion within the cell. “Fluorescent timer” protein colocalized closely with viral 3A protein within virus replication organelles. Intriguingly, infection of partially differentiated NPSCs or C2C12 myoblast cells induced the release of abundant extracellular microvesicles (EMVs) containing matured “fluorescent timer” protein and infectious virus representing a novel route of virus dissemination. CVB3 virions were readily observed within purified EMVs by transmission electron microscopy, and infectious virus was identified within low-density isopycnic iodixanol gradient fractions consistent with membrane association. The preferential detection of the lipidated form of LC3 protein (LC3 II) in released EMVs harboring infectious virus suggests that the autophagy pathway plays a crucial role in microvesicle shedding and virus release, similar to a process previously described as autophagosome-mediated exit without lysis (AWOL) observed during poliovirus replication. Through the use of this novel recombinant virus which provides more dynamic information from static fluorescent images, we hope to gain a better understanding of CVB3 tropism, intracellular membrane reorganization, and virus-associated microvesicle dissemination within the host.

## Author Summary

Enteroviruses are significant human pathogens, causing myocarditis, aseptic meningitis and encephalitis. The mechanisms of enterovirus dissemination in the host and cell-to-cell spread may be critical factors influencing viral pathogenesis. Here, we have generated a recombinant coxsackievirus expressing “fluorescence timer” protein (Timer-CVB3) which assists in following the progression of infection within the host. Unexpectedly, we observed the shedding of microvesicles containing virus in partially-differentiated progenitor cells infected with Timer-CVB3. These extracellular microvesicles (EMVs) were released in high levels following cellular differentiation, and may play a role in virus dissemination. Timer-CVB3 will be a valuable tool in monitoring virus spread in the infected host.

## Introduction

Enteroviruses (EV) are among the most common and medically-important human pathogens, and a frequent cause of central nervous system (CNS) disease [1]. Worldwide distribution of EV infection is revealed by the detection of EV-specific antibodies in the sera of approximately 75% of individuals within developed countries. For example, in 1996, approximately 10–15 million diagnosed cases of EV infection occurred in the US alone [2].

Coxsackieviruses (CV), members of the enterovirus genus, are significant human pathogens, and the neonatal central nervous system (CNS) and heart are major targets for infection. CV infection causes severe morbidity and mortality, particularly in the very young. CV infection during pregnancy has been linked to an increase in spontaneous abortions, fetal myocarditis [3], and neurodevelopmental delays in the newborn [4]. Infants infected with CV have been shown to be extremely susceptible to myocarditis, meningitis and encephalitis with a subsequent mortality rate as high as 10%. Adult infection and subsequent viral myocarditis has also been described, and a substantial proportion of patients suffering from chronic viral myocarditis eventually develop dilated cardiomyopathy, a condition underlying almost half of all heart transplants. Severe demyelinating diseases may also occur following infection, including acute disseminated encephalomyelitis [5] and acute transverse myelitis [6]. Also, a number of delayed neuropathologies have been associated with previous CV infection, including schizophrenia [7] [8], encephalitis lethargica [9], and amyotrophic lateral sclerosis [10] [11]. Previously, we have shown that CVB3 preferentially targets neural progenitor and stem cells (NPSCs) in the CNS [12] [13] [14] [15] [16] [17]. Lasting consequences have be observed in the CNS following CVB3 infection [18], and NPSCs may represent a site of virus persistence in surviving mice infected shortly after birth [19] [20]. Also, CVB3 can infect the bone marrow and reduce hematopoietic progenitor cell populations [21].

We wished to more carefully observe CVB3 infection in the context of an ongoing Type I interferon response in order to visualize the dynamics of virus dissemination simultaneously with counteracting and protective antiviral responses generated in neighboring cells. Virus dissemination within the host may be an important consideration in predicting eventual pathogenesis in the host. Although challenging, identifying the sequence of infection upon initial virus exposure could be critical in preventing the natural disease course. For example, CVB3 has been shown to target the pancreas at early time points following infection. In addition to initiating acute pancreatitis, early virus replication in the pancreas may shed additional virions which disseminate in the host eventually reaching the heart or CNS. Organ-specific expression of interferon-γ within the pancreas was previously shown to reduce initial CVB3 replication and limit acute myocarditis in the host [22]. Therefore, hindering the normal progression of virus dissemination by targeting early sites of infection may be a possible strategy to protect the host.

In order to track virus dissemination both in cultured cells and *in vivo*, we generated a recombinant CVB3 expressing “fluorescent timer” protein (Timer-CVB3). “Fluorescent timer” protein encodes a mutated form (V105A, S197T) of the red fluorescent protein, drFP583 [23]. drFP583 protein has been shown to fluoresce green immediately following translation. The red fluorescence of the native protein, drFP583, develops over a periods of a few hours following autocatalytic modification. The engineered mutations in “fluorescent timer” protein resulted in this autocatalysis process being substantially delayed and hence fluoresces green for a greater period of time following translation. Immediately after translation, the mutant “fluorescent timer” protein shows strong green fluorescence, but over a period of ∼48 hours is gradually converted to red fluorescence most likely due to a conformational change. This progressive conversion from green to red fluorescence assisted in determining the sequence of infection and virus spread in real time within the host. In general, more dynamic information regarding viral infection was obtained from static fluorescent images utilizing Timer-CVB3 thereby showing viral spread among neighboring cells.

Intriguingly, extracellular microvesicles (EMVs) containing infectious virus were readily observed in cultures of differentiated progenitor cells infected with Timer-CVB3. Many of these shed microvesicles were comprised of microtubule-associated protein light chain 3 (LC3), a protein essential for the generation of double membrane autophagosomes in the cytosol of cells [24]. Through the use of this novel recombinant virus, we hope to gain a better understanding of CVB3 tropism, spread, and pathogenesis in our animal model of infection.

## Results

### Generation and characterization of a novel recombinant CVB3 expressing Timer protein (Timer-CVB3) in cultured HeLa RW cells

**Figure 1:**
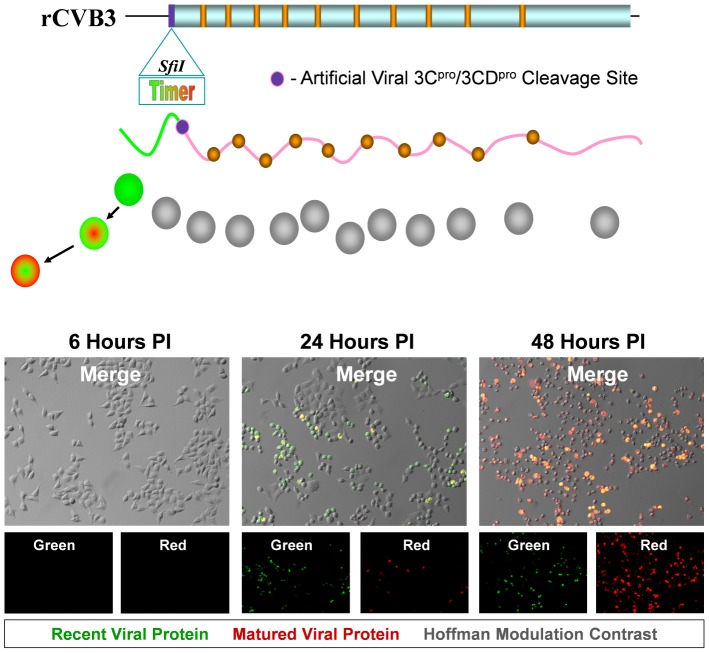
HeLa cells infected with Timer-CVB3 slowly change fluorescence from green to red. The gene for “fluorescent timer” protein was inserted into the infectious plasmid clone for CVB3 (pMKS1) which contains a unique *Sfi*1 restriction site followed by the coding sequence for a polyglycine linker and a 3C^pro^/3CD^pro^ cleavage site (shown as a purple circle) immediately downstream of the viral polyprotein start codon. Upon HeLa cell infection with recombinant CVB3 expressing “fluorescent timer” protein (Timer-CVB3), we expected the slow conversion of the green fluorescing form of timer protein to red (shown diagrammatically). HeLa cells infected with Timer-CVB3 (moi = 0.1) initially fluoresced green (recent viral protein) at 24 hours PI as determined by fluorescence microscopy. By 48 hours PI, both green and red fluorescence (matured viral protein) was observed in infected HeLa cells, although the majority of cells fluoresced brightly in the red channel.

The “fluorescent timer” protein slowly turns from green to red fluorescence allowing for temporal discrimination of recently-infected and previously-infected cells. As shown in **Figure 1**, the gene encoding Timer protein followed by a polyglycine linker and an artificial viral 3C^pro^/3CD^pro^ protease cleavage site was inserted into the backbone of our infectious CVB3 plasmid, as described for other recombinant CVB3s [25] [26]. The artificial viral 3C^pro^/3CD^pro^ protease cleavage site efficiently results in the processing of foreign proteins such as the enhanced green fluorescent protein, dsRED protein, and “fluorescent timer” protein from the adjacent viral VP4 protein [26] [27] [28]. A high-titered stock of Timer-CVB3 was generated after transfection of the infectious plasmid in to HeLa cells.

Following the infection of HeLa RW cells with Timer-CVB3 virus stock, “fluorescent timer” protein expression was observed by fluorescence microscopy. Similar to previous studies for “fluorescent timer” protein [23], the transition from green to red fluorescence was observed over the course of 48 hours post-infection (PI). After 24 hours, infected cells predominantly expressed high levels of green “fluorescent timer” protein. By 48 hours PI, infected cells fluoresced both colors, although predominantly red. These results demonstrate that Timer-CVB3 can be used as a molecular timer to mark and follow infected cells temporally. The growth kinetics and plaque size of Timer-CVB3 was found to closely match eGFP-CVB3 and dsRED-CVB3, both of which contain foreign inserts of similar size [29]. Generally, we have observed a reduction in recombinant CVB3 growth kinetics dependent upon the size of the gene insert [25] [26]. Nonetheless, recombinant CVB3s maintain their infectivity *in vivo* causing neuropathology in the neonatal mouse model [18] [19] and pancreatitis in the adult mouse model [30] [31] similar to wild type CVB3.

### HeLa cells infected with Timer-CVB3 slowly changed fluorescence from green to red as determined by flow cytometric analysis

**Figure 2:**
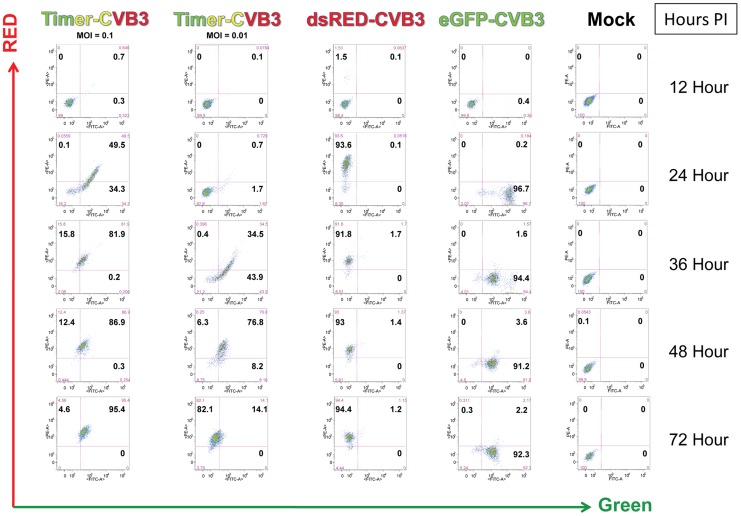
HeLa Cells infected with Timer-CVB3 slowly change fluorescence from green to red as determined by flow cytometric analysis. HeLa cells were either mock-infected or infected with eGFP-CVB (moi = 01), dsRed-CVB3 (moi = 0.1), or Timer-CVB3 (moi = 0.01 or 0.1). After the indicated times post-infection, the cells were isolated, centrifuged and resuspended in a solution of 4% paraformaldehyde in 1× PBS for fixation overnight. The cells were then centrifuged and resuspended in 0.1% BSA/1× PBS, and the cell solutions were stored at 4°C until analyzed by flow cytometry. Quadrants were set based in mock-infected control samples.

HeLa cells were infected with Timer-CVB3, dsRED-CVB3, or eGFP-CVB3 and monitored by flow cytometry (**Figure 2**), *a more* sensitive and quantitative readout of viral protein expression allowing analyses at the single cell level. As expected, dsRED-CVB3 and eGFP-CVB3-infected HeLa cells expressed high levels of their respective fluorescent reporter proteins as early as 24 hours PI. However, Timer-CVB3-infected HeLa cells slowly changed fluorescence from green to red over 72 hours PI (moi = 0.01). HeLa cells infected with a greater moi (moi = 0.1) showed an increase in the number of green and red fluorescing cells at 24 hours PI, as compared to HeLa cells infected at the lower moi. Based on the kinetic data of live infected cultures observed by fluorescence microscopy shown in **Figure 1**, we might have anticipated the complete conversion of “fluorescent timer” protein from green to red in Timer-CVB3-infected HeLa cells harvested at early time points (for example, 24 hours PI) due to the delay between harvesting and ensuing analysis by flow cytometry. However, cells were fixed in 4% paraformaldehyde shortly after harvesting and prior to flow cytometry. Subsequently, the gradual change in fluorescence for Timer-CVB3-infected HeLa RW cells could be arrested in the presence of 4% paraformaldehyde, suggesting that the tertiary structure of “fluorescent timer” protein is stabilized by formaldehyde cross-linking reactions.

### Timer-CVB3 plaque progression and viral spread in HeLa cells and NPSCs

**Figure 3:**
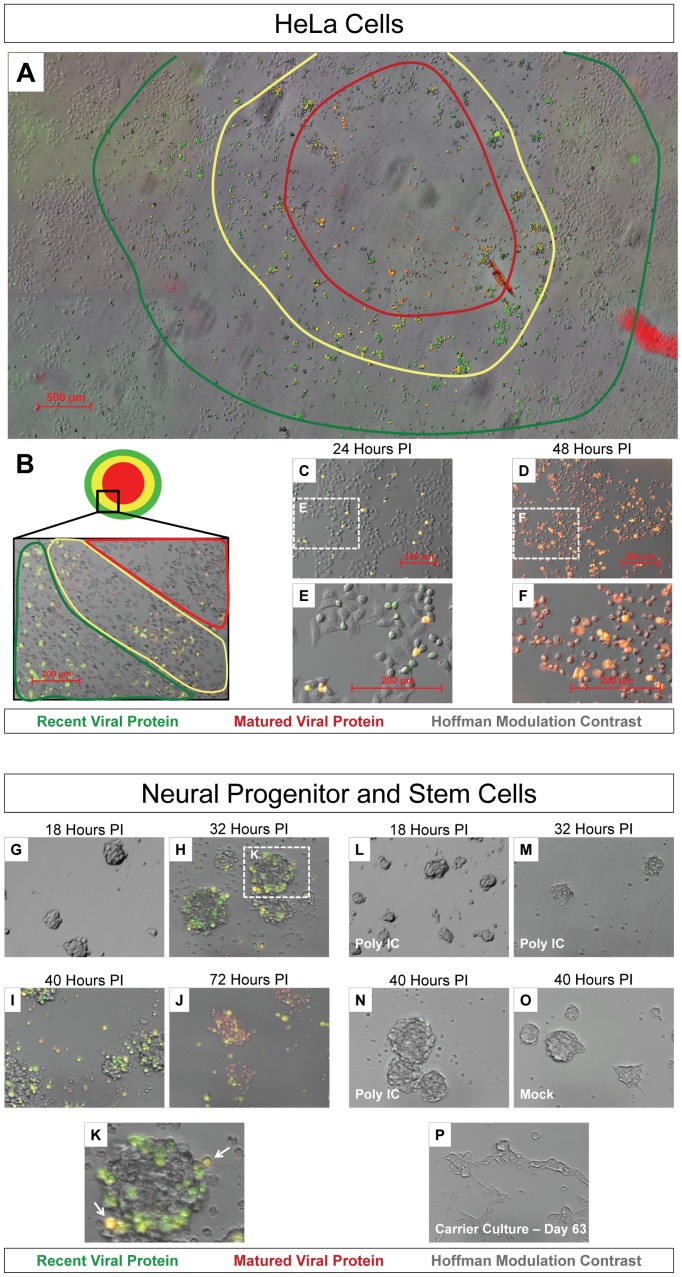
Timer-CVB3 plaque progression and viral spread in HeLa cells and NPSCs. *(A)* The conversion of “fluorescent timer” protein was observed in an assembled composite of overlapping 5× fluorescence microscopic images during plaque formation on HeLa cells infected with Timer-CVB3 and overlayed with 0.3% agar. A “bulls-eye” pattern was seen by fluorescence microscopy whereby matured viral protein (red region) was detected in early infected cells located in the center of the plaque, and recent viral protein (green region) was detected in cells located further from the plaque center. *(B) Higher magnification revealed Timer-CVB3 plaque progression, viral spread, and cytopathic effects as illustrated by red, yellow and green regions within the plaque.* *(C), (E)* HeLa cells grown in culture and infected with Timer-CVB3 at 24 hours PI fluoresced yellow and green (recent viral protein). *(D), (F)* HeLa cells infected with Timer-CVB3 at 48 hours PI fluoresce yellow and red (matured viral protein). *(G), (H), (I), (J), and (K)* Progression of NPSCs infected with Timer-CVB3 was observed by fluorescence microscopy. *(L), (M), (N), (O)* NPSCs pretreated with poly IC showed reduced infection with Timer-CVB3. *(P)* Carrier-state NPSCs infected with Timer-CVB3 failed to express detectable levels of “fluorescent timer” protein.

By standard plaque assay, Timer-CVB3 plaques were expected to reveal a “bull's-eye” pattern by fluorescence microscopy at 44 hours PI, whereby initial infection is represented by red fluorescence and newly-infected cells via cell-to-cell spread is represented by green fluorescence. The progression of infection was followed over time in HeLa cells infected with Timer-CVB3 and overlayed with agar which hindered virion dispersal in the culture medium. As expected, a gradual change in fluorescence was observed for a single viral plaque in a composite of fluorescence images taken from agar-overlayed infected-HeLa cells at 44 hours PI (**Figure 3A**). Higher magnification of a segment of the viral plaque revealed a progressive shift from red to yellow to green cells away from the plaque center (**Figure 3B**). Cells showing cytopathic effects were also evident and these cells exhibited reduced or absent levels of “fluorescent timer” protein. In contrast, HeLa cells infected with Timer-CVB3 (moi = 0.1) and grown in the absence of an agar plug showed diffuse infection and virus spread, although individual cells fluoresced green and yellow at 24 hours PI (**Figure 3C** and **Figure 3E**). By 48 hours PI, the majority of HeLa cells grown in complete media in the absence of an agar plug fluoresced red (**Figure 3D** and **Figure 3F**).

Neural progenitor and stem cells (NPSCs) grown in culture as free-floating clusters of stem cells or “neurospheres” were recently shown to be highly susceptible to CVB3 infection [12] [19] [28]. We anticipated that the outer shell of stem cells first becomes infected, after which infection extends inward by cell-to-cell spread of CVB3. Therefore, NPSCs were infected with Timer-CVB3 (moi = 0.1), and the progression of infection was monitored over time by fluorescence microscopy (**Figure 3G**, **Figure 3H**, **Figure 3I**, and **Figure 3J**). By 32 hours PI, Timer protein expression outlined the progressive infection of the outer (yellow-fluorescing, white arrow) and inner stem cells (green fluorescing) within the neurosphere (**Figure 3K**). By 72 hours PI, red and yellow fluorescing cells were seen within disrupted neurospheres, and signs of cytopathic effects were readily evident. Intriguingly, pretreatment of NPSCs with poly IC, a synthetic analogue of double-stranded RNA known to interact with toll-like receptor 3, reduced the progression of infection as determined by “fluorescent timer” protein expression (**Figure 3L**, **Figure 3M**, and **Figure 3N**). These results suggest that NPSCs respond to immunostimulatory molecules and mount a protective antiviral response following CVB3 infection. No “fluorescent timer” protein signal was observed in mock-infected NPSCs by fluorescence microscopy (**Figure 3O**).

Our recent results suggest that CVB3 established a carrier-state infection in NPSCs [12] [29]. Carrier-state infection in NPSCs may be the result of continuous virus replication, or alternatively, sporadic reactivation of virus. We expect that the utilization of Timer-CVB3 will be useful in distinguishing these two models of carrier-state infection by examining “fluorescent timer” protein expression. However, timer protein expression was below detection limits despite the presence of infectious virus (as determined by plaque assay) in Timer-CVB3-infected NPSC carrier state cultures (**Figure 3P**).

### IFN-β or poly IC treatment restricted the progression of Timer-CVB3 infection in HeLa cells at low multiplicities of infection

Host cells infected with RNA viruses induce protective molecules such as MAVS through RIG-I and MDA5 activation which act as pattern recognition receptors [32]. The host cell undergoes an antiviral state and produces Type I interferons such as interferon-α (IFN-α) and IFN-β which act to protect neighboring cells. The Type I IFN response enables neighboring cells to express protective molecules such as RNase L which limits the spread of viral infection.

**Figure 4:**
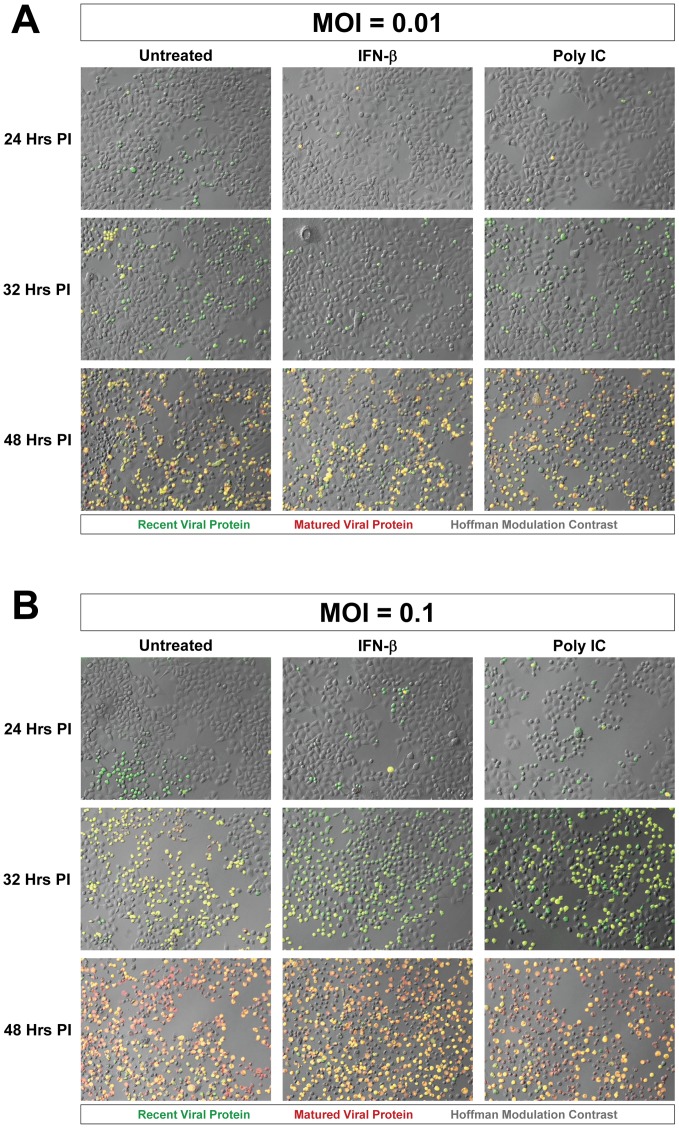
Progression of Timer-CVB3 infection in HeLa cells pretreated with IFN-β or Poly IC. *(A)* At low moi (moi = 0.01), more green cells were observed in untreated HeLa cells as compared to either of the treatment conditions at 24 hours PI. At 32 hours PI, more yellow cells were observed in untreated cultures. At 48 hours PI, all cultures contain a mixture of red, green, yellow fluorescing cells. *(B)* At the higher moi (moi = 0.1), the cultures appear to be very similar across the three different conditions for each of the three time points.

We inspected the progression of Timer-CVB3 infection in HeLa cells activated by the Type I IFN response to determine the pattern of virus spread within neighboring cells in the context of an active host antiviral response. Untreated HeLa cells, or HeLa cells treated with IFN-β or Poly IC were infected with Timer-CVB3 at a low (moi = 0.01) or greater moi (moi = 0.1), and the progression of infection was monitored by fluorescence microscopy (**Figure 4**). Untreated HeLa cells infected with Timer-CVB3 at a low moi initially fluoresced green at 24 hours PI (**Figure 4A**). By 32 hours PI, yellow and green cells appeared, and the majority of HeLa cells fluoresced yellow or red by 48 hours PI. In both IFN-β and poly IC-treated HeLa cells infected at a low moi, infection appeared delayed at 24 hours PI. Also, fewer infected cells were observed at 32 hours PI for IFN-β -treated HeLa cells, and these cells predominantly fluoresced green. By 48 hours PI, both IFN-β and poly IC-treated HeLa cells fluoresced yellow and red similar to untreated HeLa cells. These results suggest that the Type I IFN response partially protected HeLa cells from a low inoculum of Timer-CVB3 and initially controlled the progression of infection. In contrast, little protection was observed in HeLa cells infected with a higher inoculum of Timer-CVB3 (**Figure 4B**). This observable yet modest protection against CVB3 may be partly due to the relatively weak Type I IFN response induced by HeLa cells, and also the ability of CVB3 to inactivate key antiviral molecules. For example both MAVS and TRIF, known to be critical components of the innate immune response, have been shown to be cleaved in CVB3-infected cells [33].

**Figure 5:**
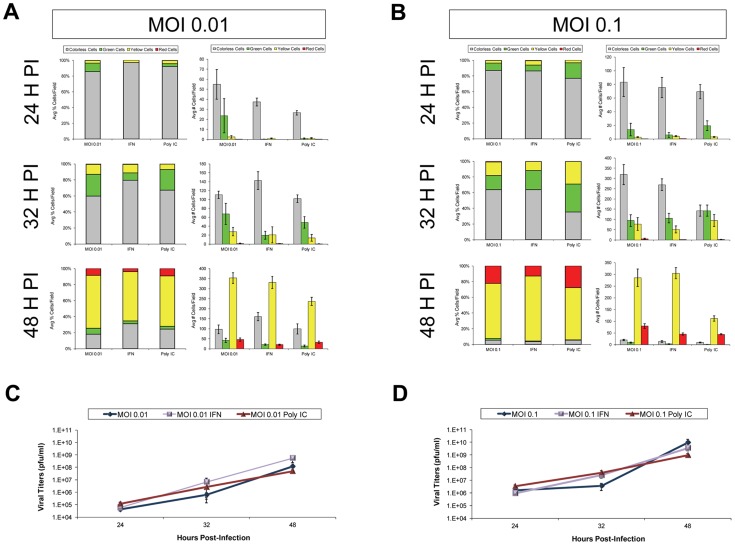
IFN-β or poly IC treatment restricted the progression of Timer-CVB3 infection in HeLa cells at low multiplicities of infection. *(A)* HeLa cells treated with IFN-β or Poly IC showed fewer signs of cytopathic effects and fewer green cells by fluorescence microscopy following infection with Timer-CVB3 as compared to untreated cultures at 24 hours PI. For HeLa cells given a relatively low amount of viral inoculum (moi = 0.01), untreated cultures showed the highest percentage of green cells. By 32 hours PI, the number of cells exhibiting cytopathic effects was similar across the three conditions, and the percentage of cells expressing green “fluorescent timer” protein was similar between the untreated and the poly IC-treated cultures. In contrast IFN- β -treated HeLa cells continued to show reduced numbers of green cells. By 48 hours PI, the majority of the infected HeLa cells expressed both early and late viral proteins (yellow signal), and no significant differences were observed between the three conditions. *(B)* The effects of IFN- β and poly IC were minimized at higher moi. *(C)*, *(D)* Viral titers were similar between treatments at both the low and high moi.

The results showing a limited reduction in Timer-CVB3 progression in HeLa cells infected with a low or greater viral inoculum were quantified using ImageJ analysis (**Figure 5A** and **Figure 5B**, respectively). Despite the delay of Timer-CVB3 infection as determined by fluorescence microscopy following IFN-β or poly-IC treatment, no significant difference in viral titers was observed over time in HeLa cells infected with a low or high viral inoculum (**Figure 5C** and **Figure 5D**, respectively). These results suggest that monitoring the progression of infection using “fluorescent timer” protein may be a more sensitive method of determining the efficacy of an antiviral response within infected cells.

Separately, HeLa cells were infected with Timer-CVB3 at moi = 0.01 or 0.1 and treated with two concentrations of ribavirin (10 µg/mL and 100 µg/mL, respectively; **Figure S1**). A clear step-wise reduction in cytopathic effects, and “fluorescent timer” protein conversion and expression levels was observed in HeLa cells (moi = 0.01) treated with increasing amounts of ribavirin at 24, 32, and 48 hours PI (**Figure S1A**). Although less striking, HeLa cells infected at a higher moi (moi = 0.1) and treated with two concentrations of ribavirin also showed delayed cytopathic effects, and reduced levels of “fluorescent timer” protein conversion and expression levels (**Figure S1B**). These results were quantified using ImageJ analysis to show a substantial difference in the percentage of ribavirin-treated HeLa cells expressing “fluorescent timer” protein over time (**Figure S2A** and **Figure S2B**). Also, a significant delay was observed in the conversion of recent viral protein to mature viral protein at 24, 32, and 48 hours PI as compared to untreated HeLa cells. In addition, a step-wise reduction in Timer-CVB3 viral titers was observed in HeLa cells treated with ribavirin, although the reduction was less dramatic in HeLa cells infected with a higher inoculum of virus (**Figure S2C** and **Figure S2D**).

### Timer-CVB3 infection of mixed cell cultures

We previously described the ability of CVB3 to preferentially replicate in undifferentiated NPSCs [12]. However, a limited amount of viral replication was also observed in differentiated NPSCs comprising of a mixture of progenitor cells, and early neuronal, astrocytic, and oligodendritic cell lineages. Also, the relationship between CVB3 infection and autophagy in differentiated NPSCs proved to be quite distinct and unique as compared to either undifferentiated NPSCs or traditional cell lines [28]. We wished to follow the progression of Timer-CVB3 infection in mixed cell lineages comprising differentiated NPSC cultures. For example, preferential infection of progenitor cells might be anticipated, and delayed infection of cell lineage-committed cells might be revealed by a shift in “fluorescent timer” protein expression pattern whereby progenitor cells fluoresce red and more lineage restricted cells fluoresce green at a particular time point following infection in these mixed cultures. Therefore, NPSCs were differentiated for 5 days and infected with Timer-CVB3.

**Figure 6:**
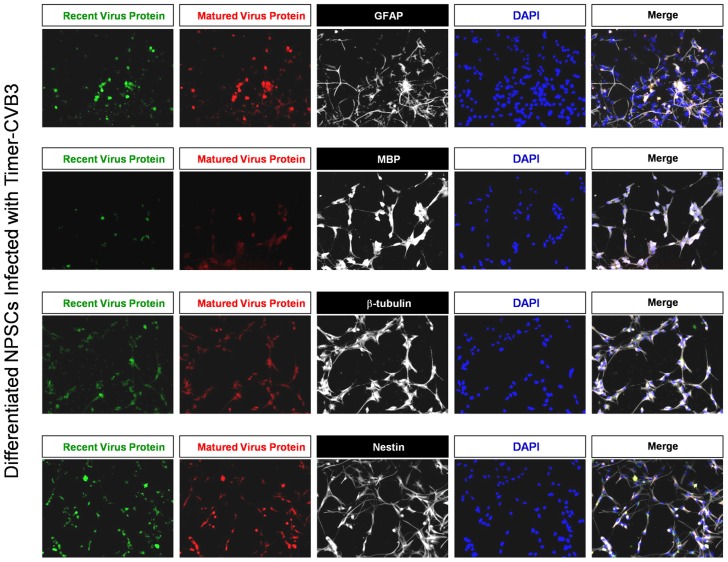
Inspection of recent and matured virus protein expression in differentiated NPSCs infected with Timer-CVB3. NPSCs cultures were differentiated for five days, and then infected with Timer-CVB3 for three days, followed by immunostaining for lineage markers (white signal). “Fluorescent timer” protein produced early on (matured virus protein) marked cells which were infected early on. Newly infected cells were distinguished by green fluorescence (recent viral protein).

After 48 hours PI, cells were fixed and stained for progenitor (nestin^+^), neuronal (neuronal beta-tubulin III^+^), astrocytic (GFAP^+^), and oligodendritic (MBP^+^) cell lineage markers (**Figure 6**). Four-color fluorescence microscopy identified recent and matured virus protein expression in combination with each cell lineage marker, and DAPI was utilized to identify cell nuclei. The distribution of recent and matured virus protein appeared similar in nestin^+^ progenitor cells and in the three cell lineages, suggesting that progenitor cells and each cell lineage were equally susceptible to Timer-CVB3 infection upon initial infection.

### Shedding of EMVs containing viral protein following Timer-CVB3 infection of differentiated cells

Previously, we described cellular blebbing in partially differentiated NPSCs infected with eGFP-CVB3 by Hoffman modulation contrast microscopy [12]. Interestingly, cell-associated and cell free microvesicles comprising recent and matured virus protein were readily observed at higher magnification in differentiated NPSCs infected with Timer-CVB3 (data not shown). We hypothesized that these cell-associated and cell-free microvesicles were reminiscent of the cellular blebbing phenomena observed in infected differentiated NPSCs described previously.

**Figure 7:**
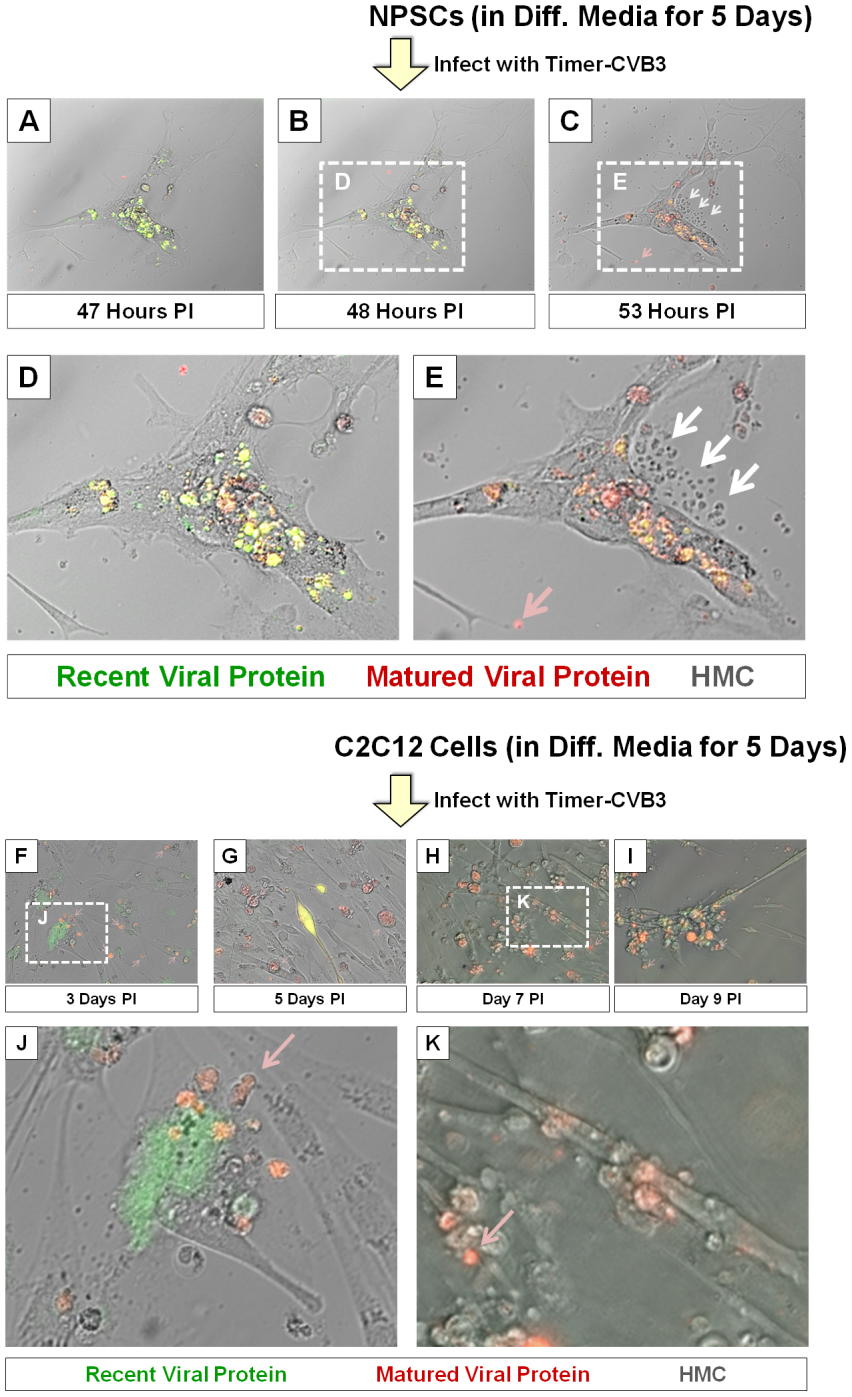
Shedding of EMVs containing viral protein following Timer-CVB3 infection of differentiated cells. *(A–C)* “Fluorescent timer” protein changed from green to red over the span of 6 hours in differentiated NPSCs infected with Timer-CVB3. *(D)*, *(E)* Digital magnification of *(B)* and *(C)*, respectively, revealed the shedding of EMVs (white arrows) some containing matured “fluorescent timer” protein (pink arrow) between 48 and 53 hours post infection. *(F–I)* Abundant EMVs containing matured “fluorescent timer” protein were observed in differentiated C2C12 myoblast cells infected with Timer-CVB3. *(J)* Digital magnification demonstrated the presence of EMVs containing matured “fluorescent timer” protein (pink arrow) near recently-infected cells in green at 3 days PI. *(K)* By 7 days PI, EMVs containing matured “fluorescent timer” protein (pink arrow) were observed near cells with signs of cytopathic effects.

In order to better observe cell-associated microvesicles following CVB3 infection, time-lapse videos were constructed from merged fluorescence images (recent and matured virus protein with HMC) of differentiated NPSCs infected with Timer-CVB3. Merged images were taken every 15 minutes for 7 hours to construct time-lapse videos (**Video S1**). The conversion of “fluorescent timer” protein was monitored over time in Timer-CVB3-infected cells which showed in parallel extensive intracellular membrane remodeling during infection. We hypothesized that the appearance of intracellular microvesicles expressing “fluorescent timer” protein represented the emergence of virus replication organelles recently described by others for CVB3 [34]. Intriguingly, the presence of numerous extracellular microvesicles (EMVs) was also identified in supernatants of the cell culture (**Figure 7**), although some of these particles may represent cellular debris. Many EMVs contained matured virus protein rather than recent virus protein, as evident by the detection of red “fluorescing timer” protein. Furthermore, some shed microvesicles containing viral protein appeared to attach and remain stationary on uninfected neighboring cells. Images from the time-lapse video of differentiated NPSCs infected with Timer-CVB3, or separately, differentiated C2C12 myoblast cells infected with Timer-CVB3, are shown in **Figure 7**. The progression of infection in differentiated NPSCs as determined by “fluorescent timer” protein was captured in revealing images taken at 47, 48, and 53 hours PI (**Figure 7A**, **Figure 7B**, and **Figure 7C**, respectively). Higher magnification showed the compartmentalization of distinct intracellular viral replication organelles of varying fluorescence color following Timer-CVB3 infection (**Figure 7D**), and the accumulation of EMVs (**Figure 7E**, white arrows) some of which fluoresced red (**Figure 7E**, pink arrow). We suspect that many additional shed microvesicles contain virus protein albeit at low or undetectable levels as judged by fluorescence microscopy.

Of note, differentiated C2C12 myoblast cells infected with Timer-CVB3 also gave rise to red fluorescing EMVs, beginning at 3 days, and also at 5, 7, and 9 days PI (**Figure 7F**, **Figure 7G**, **Figure 7H**, and **Figure 7I**, respectively). Mouse C2C12 cells are a myoblast cell line capable of differentiation into myocyte cells. Higher magnification revealed the presence of red fluorescing microvesicles near a green fluorescing cell, suggesting a new infection event had taken place (**Figure 7J**, pink arrow). By 9 day PI, high magnification showed red fluorescing microvesicles near more defined myocyte cells with signs of cytopathic effects (**Figure 7K**, red arrow). The presence of numerous red EMVs in two contrasting models of infected differentiating progenitor cells suggests that the differentiation process plays a role in their formation and release from the target cell.

**Figure 8:**
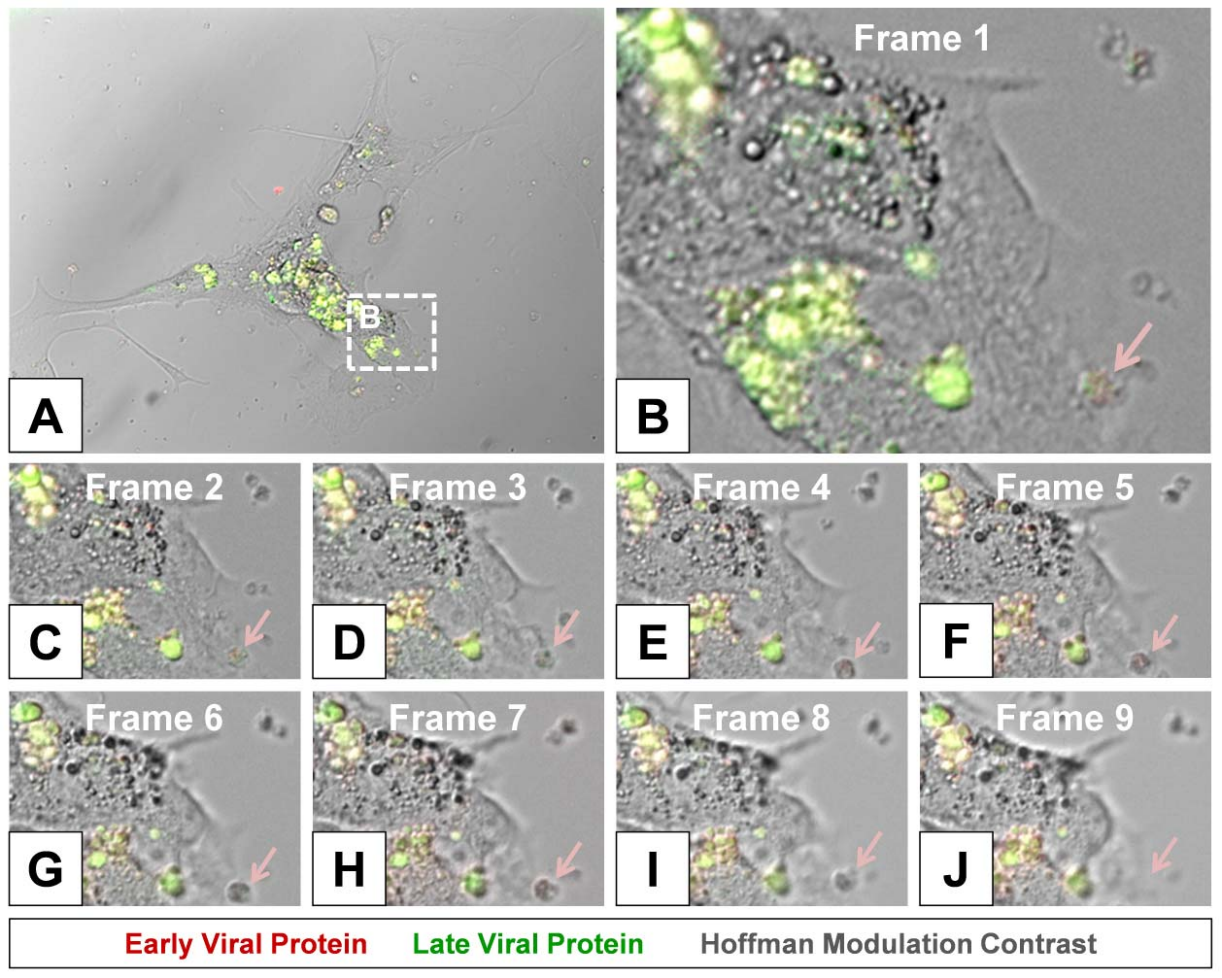
Time lapse photography frames showing microvesicle egress from a Timer-CVB3-infected cell. *(B–I)* Close inspection of image *(A)* taken at 15 minute intervals in differentiated NPSCs infected with Timer-CVB3 and observed during a six hour span revealed intracellular membrane reorganization (first 8 frames shown). *(J)* An intracellular microvesicle expressing matured “fluorescent timer” protein and formed following Timer-CVB3 infection was released from the infected cell (frame 9; pink arrow).

High magnified image frames from the first 90 minutes of the time-lapse video shown in **Video S1** revealed the progression of intracellular membrane remodeling and “fluorescent timer” protein expression reminiscent of CVB3 replication organelles described previously by others [34] (**Figure 8**). As shown in frame 9, the loss of a red fluorescing intracellular membrane complex with matured virus protein represented potential microvesicle egress from the infected cell (**Figure 8B–J**; red arrow). A high magnification time-lapse video of this area (**Video S2**) and other areas (**Video S3** and **Video S4**) also revealed the possible ejection of additional microvesicles from the infected cell.

### Colocalization of viral 3A protein and “fluorescent timer” protein in viral replication organelles within the cell

**Figure 9:**
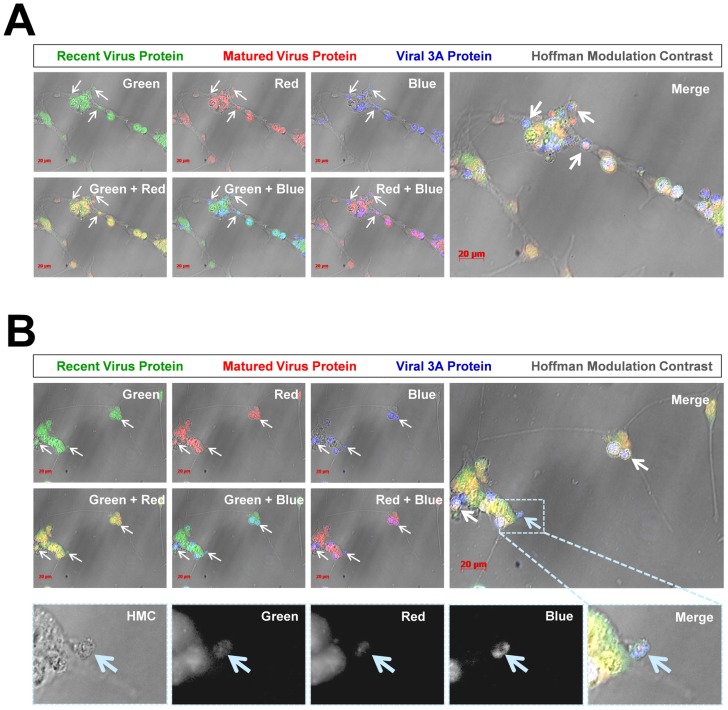
Colocalization of “fluorescent timer” protein and viral 3A protein in NPSCs infected with Timer-CVB3. NPSCs were differentiated for five days and infected with Timer-CVB3 for four days. Infected cells were fixed and immunostaining for viral 3A protein (blue) or observed for native “fluorescent timer” protein expression. Optical sections of stained cells were evaluated for viral protein colocalization utilizing a Zeiss Axio Observer with ApoTome Imaging System. (A) Viral 3A protein colocalized with both recent and matured “fluorescent timer” protein. Also, viral 3A protein was preferentially observed within intracellular microvesicles (white arrows) associated with the cellular membrane. (B) An additional field of infected cells was evaluated, and viral 3A protein colocalized with both recent and matured “fluorescent timer” protein, although the distribution of “fluorescent timer” protein appeared more widespread and viral 3A protein was preferentially associated with intracellular microvesicles. Closer inspection of a single microvesicle (High magnification images highlighted in the dashed cyan box) revealed colocalization of viral 3A protein with both recent and matured “fluorescent timer” protein (cyan arrow; black and white images).

We inspected the location of ‘fluorescent timer” protein with an authentic CVB3 protein within differentiated NPSCs (**Figure 9**; two representative images shown). An antibody against viral 3A protein was utilized for colocalization experiments since this viral protein has previously been shown to reorganize the host secretory trafficking pathway and facilitate the recruitment of host proteins necessary to form specialized organelles critical for plus-strand RNA virus replication [34]. Partially differentiated NPSCs were infected with Timer-CVB3 for 4 days and fixed with 2% paraformaldehyde. Optical sections of fluorescently stained cells using the viral 3A antibody revealed a close colocalization with both recent and matured “fluorescent timer” protein in differentiated NPSCs utilizing a Zeiss Axio Observer with ApoTome Imaging System (**Figure 9A** and **Figure 9B**; white arrows). Intriguingly, the viral 3A protein was preferentially detected in cell-associated vesicles. Both recent and matured “fluorescent” protein was also detected in cell-associated vesicles, although the signal was more diffuse as compared to viral 3A protein. Closer inspection of a cell-associated microvesicle (high magnification) revealed the presence of recent (green), matured (red), and viral 3A protein (blue) (**Figure 9B**; cyan arrow). These results suggest that “fluorescent timer” protein was preferentially found in regions of the infected cell where active viral replication organelle complexes might be expected.

### Detection of LC3 protein in shed microvesicles containing viral protein

**Figure 10:**
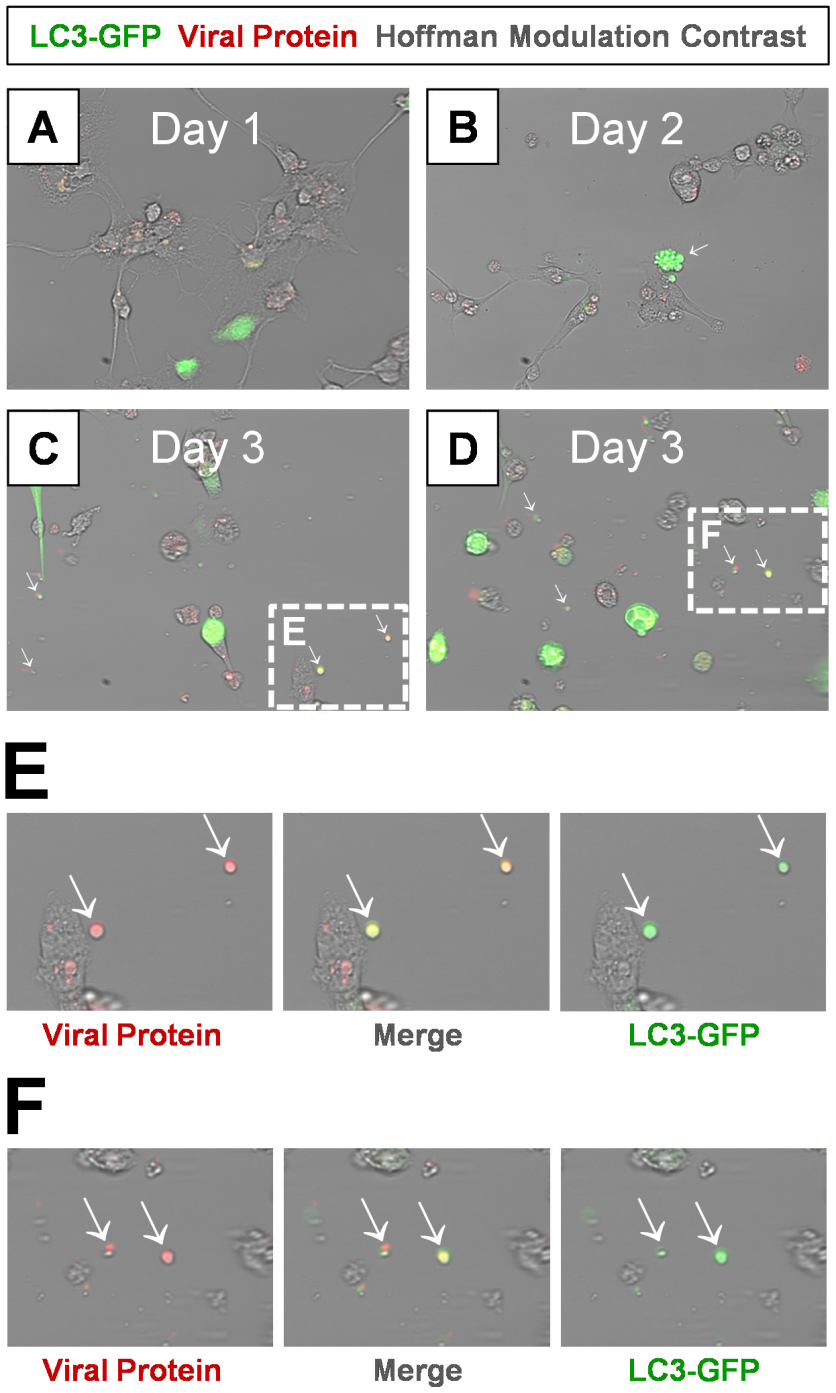
Detection of LC3 protein in shed EMVs containing viral protein. Differentiated NPSCs transduced with adeno-LC3-GFP were infected with dsRED-CVB3 (moi = 0.1) and observed by fluorescence microscopy up to 3 days PI. *(A)* Transduced NPSCs (green) and viral protein expression (red) were readily observed by day 1 PI. *(B)* Differentiated NPSCs infected with virus showed signs of cellular blebbing by day 2 PI (white arrow). *(C–D)* Abundant shed EMVs were readily observed by day 3 PI. EMVs (white arrows) contained viral protein (red) and expressed a marker for autophagosomes (LC3-GFP, green). *(E–F)* Higher magnification of *(C)* and *(D)* showed colocalization of viral protein and LC3-GFP in shed EMVs.

Previous studies with poliovirus described the possible contribution of the autophagic pathway to virus egress from the host cell [35]. Therefore, we transduced differentiated NPSCs with an adenovirus expressing LC3-GFP as described previously [28], and followed autophagosome formation following infection with dsRED-CVB3 (**Figure 10**). By day 1 PI, cells expressed LC3-GFP and detectable levels of virus protein (red) (**Figure 10A**). Cellular blebbing associated with LC3-GFP signal was seen in infected cells as early as day 2 PI (**Figure 10B**; white arrow). By day 3 PI, abundant EMVs were observed in culture containing both viral material (red signal) and LC3-GFP protein (green signal) (**Figure 10C** and **Figure 10D**; white arrows). Higher magnification further revealed the size and structure of representative shed microvesicles which contained both viral material and LC3-GFP (**Figure 10E** and **Figure 10F**; white arrows). These results suggest that the observed EMVs following CVB3 infection arise from the autophagy pathway shown previously to be activated following infection [36] [37] [30] [38] [39] [40] [28].

### Shed microvesicles harbor infectious virus

**Figure 11:**
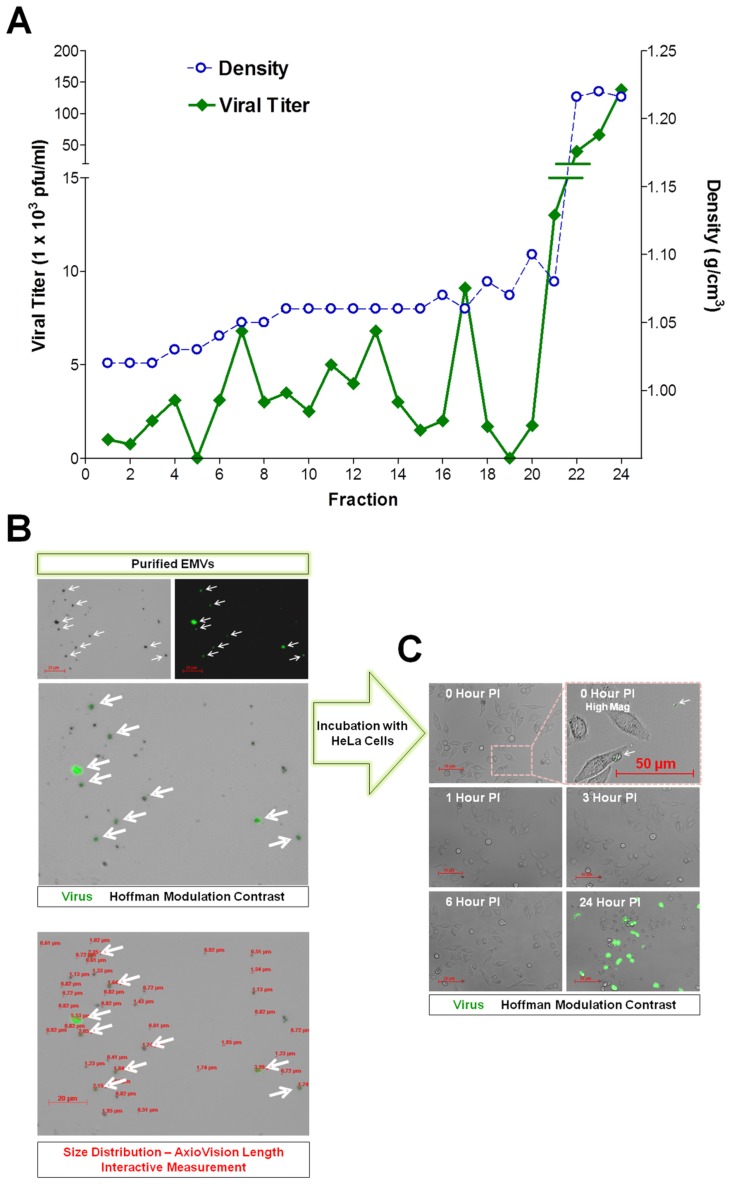
Purified EMVs isolated from infected progenitor cells contained infectious virus. *(A)* Supernatants from differentiated C2C12 cell cultures infected with eGFP-CVB3 were collected at day 3 PI and purified by Isopycnic gradient centrifugation. Buoyant density of infectious, membrane-associated EMVs released by C2C12 cells were observed within a range of 1.04–1.10 g cm^−3^. In contrast, a separate peak of infectious virus was observed at a density expected for non-enveloped virions (1.22 g cm^−3^). *(B)* Shed microvesicles isolated from differentiated C2C12 cells infected with eGFP-CVB3 were collected at day 3 PI and purified utilizing the Exoquick-TC polymer-based exosome precipitation kit. Purified EMVs were resuspended in 1× DMEM and inspected by fluorescence microscopy for viral protein (eGFP) and size distribution using the Length Interactive Measurement feature in AxioVision software. *(C)* Purified EMVs were added to HeLa cell cultures, and virus protein expression was followed by fluorescence microscopy over 24 hours PI.

Differentiated C2C12 were infected with eGFP-CVB3 for three days, and virus particles within supernatants were resolved in isopycnic iodixanol gradient fractions (**Figure 11A**). Fractions #22-24 contained infectious virus particles at a density expected for picornavirus virions (1.22 g cm^−3^). However, significant levels of infectious virus were also observed in low density fractions (Fractions #6-21) consistent with membrane association (1.04–1.10 g cm^−3^). The amount of infectious virus associated with low density fractions (Fractions #6-21) represented a significant percentage (approximately 21.5%) of the total infectious virus identified for all fractions. Also, the relatively wider range of low density fractions containing infectious virus suggested a broader density spectrum of membrane-associated virus, as compared to enveloped hepatitis A viruses resembling exosomes of a more restricted range of low density (1.06–1.10 g cm^−3^).

EMVs were also isolated from infected C2C12 supernatants utilizing Exoquick-TC polymer-based exosome precipitation kit. Purified EMVs were resuspended in 1× PBS and inspected by fluorescence microscopy for viral protein (eGFP) and size distribution using the Length Interactive Measurement feature of AxioVision software (**Figure 11B**). Numerous microvesicles expressing high levels of eGFP were identified, and particles were quite diverse in size ranging between 0.51–5.53 µm in diameter (Ave = 1.31 µm; Median = 0.92 µm; SD ±0.96), although microvesicles smaller than 0.5 µm were also shown to be present by transmission electron microscopy. The diversity in EMV size and shape might also explain the relatively wide range of low density iodixanol fractions containing infectious virus, as shown in **Figure 11A**. We tested the ability of purified EMVs to infect HeLa cells following a one hour incubation (**Figure 11C**). eGFP^+^ EMVs added to HeLa cells were visualized in the cultures shortly before (0 Hour PI) and after (1 Hour PI) the incubation period (**Figure 11C**; High Mag). By 3 hours PI, eGFP^+^ EMVs were no longer observed presumably due to fusion of the EMVs with HeLa cells and dilution of viral protein signal. By 24 hours PI, high levels of viral protein expression and cytopathic effects were readily seen in HeLa cells. These results suggest that the kinetics of EMV-associated CVB3 infection in HeLa appear similar to free infectious virus described previously for eGFP-CVB3 [26].

### Relative levels of infectious virus associated with purified EMVs and supernatant fractions

**Figure 12:**
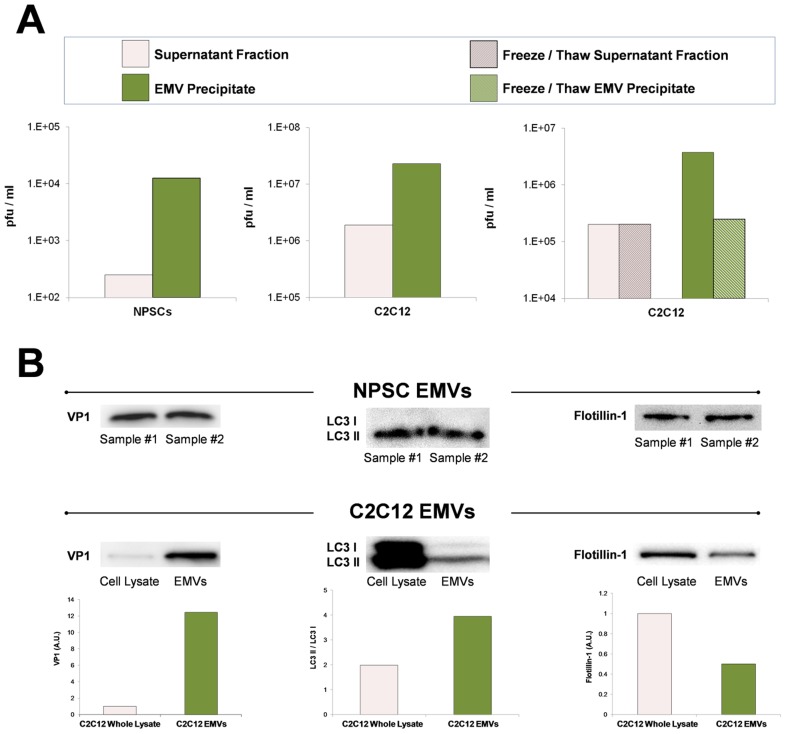
Purified EMVs isolated from infected progenitor cells expressed VP1, LC3 II and flotillin-1 proteins. Shed microvesicles isolated from differentiated C2C12 or NPSC cell cultures infected with eGFP-CVB3 were collected at day 3 PI and purified utilizing the Exoquick-TC polymer-based exosome precipitation kit. *(A)* Purified EMVs from differentiated C2C12 and NPSC cultures were resuspended with 1× PBS, and infectious virus was quantified by standard plaque assay. Levels of infectious virus in EMV precipitates (green bar) were compared to supernatant fractions (pink bar) obtained during the EMV purification procedure. Alternatively, EMV precipitates from infected differentiated C2C12 cells (green hatched bar) and supernatant fractions (pink hatched bar) were freeze/thawed 3× prior to virus titer quantification. *(B)* Purified EMVs were inspected by western analysis for the presence of coxsackievirus viral protein 1 capsid (VP1), LC3 I/LC3 II, and flotillin-1. The levels of VP1 and flotillin-1 were normalized to total protein levels quantified by bicinchoninic assay. The ratio of LC3 II to LC3 I was determined for EMVs and infected C2C12 cell lysates.

Differentiated C2C12 or NPSCs were infected with eGFP-CVB3 for three days, and EMVs were isolated utilizing Exoquick-TC polymer-based exosome precipitation kit. EMV precipitates and supernatant fractions from differentiated C2C12 and NPSC cultures were also examined for levels of infectious virus (**Figure 12A**). Purified EMVs resuspended with 1× DMEM at an equal volume compared to supernatant fractions were inspected by standard plaque assay. Intriguingly, EMV precipitate fractions for both differentiated C2C12 and NPSC cultures (**Figure 12A**; green bars) comprised a greater concentration of infectious virus compared to the supernatant fraction (**Figure 12A**; pink bars) utilizing the ExoQuick-TC isolation procedure. Freeze/thaw treatment of EMVs substantially reduced the amount of infectious virus (**Figure 12A**; green hatched bar). In contrast, freeze/thaw treatment of the supernatant fraction showed no reduction in viral titers (**Figure 12A**; pink hatched bar). These results suggest that infectious virus was preferentially associated with shed microvesicles released from differentiated C2C12 and NPSC cultures. Also, a portion of virus associated with EMVs may reflect immature particles requiring EMVs for infectivity [41], or may indicate the presence of EMV-associated viral RNA which remains infectious following microvesicle-assisted fusion of uninfected target cells. We suspect that the relative percentage of infectious virus associated with low density iodixanol gradient fractions shown in **Figure 11A** might be under-represented due to a reduction in viral titers following freeze-thaw of EMVs within the collected fractions. Nevertheless, the two independent methodologies utilized to purify EMVs indicate that a substantial amount of infectious CVB3 may comprise a membrane-associated form.

### Shed microvesicles express viral protein 1 (VP1), high levels of LC3 II, and flotillin-1

Purified EMVs were also inspected by western analysis for the presence of CVB3 viral protein 1 capsid protein (VP1), LC3 I and II, and flotillin-1 (**Figure 12B**). LCII (lipidated form of LC3) studs the inner and outer autophagosome membrane and reflects autophagic activity in cells [24]. Flotillin-1 is a caveolae-associated membrane protein identified as a common marker on exosomes [42], and at compartments of the endocytic and autophagosomal pathways. As shown in **Figure 12B**, high levels of VP1 were identified in purified EMVs isolated from both differentiated NPSC and C2C12 infected with eGFP-CVB3. Also, both NPSC and C2C12 EMVs were comprised mainly of LC3 II protein, and high levels of flotillin-1 protein were also observed by western analysis. The preferential detection of LC3 II by western blotting might be expected if EMVs originate as double membrane autophagosomes, fuse with the plasma membrane, and are released as single membrane vesicles [43] [35]. Taken together, these results verified the presence of viral proteins and infectious virus in EMVs, and demonstrate possible autophagosome-mediated exit of shed infectious vesicles reminiscent of the “autophagosome-mediated exit without lysis” pathway (AWOL) described previously by Jackson and colleagues for poliovirus infection [35].

### Transmission electron microscopy of EMVs reveals the presence of virus-like particles enclosed in membranes

**Figure 13:**
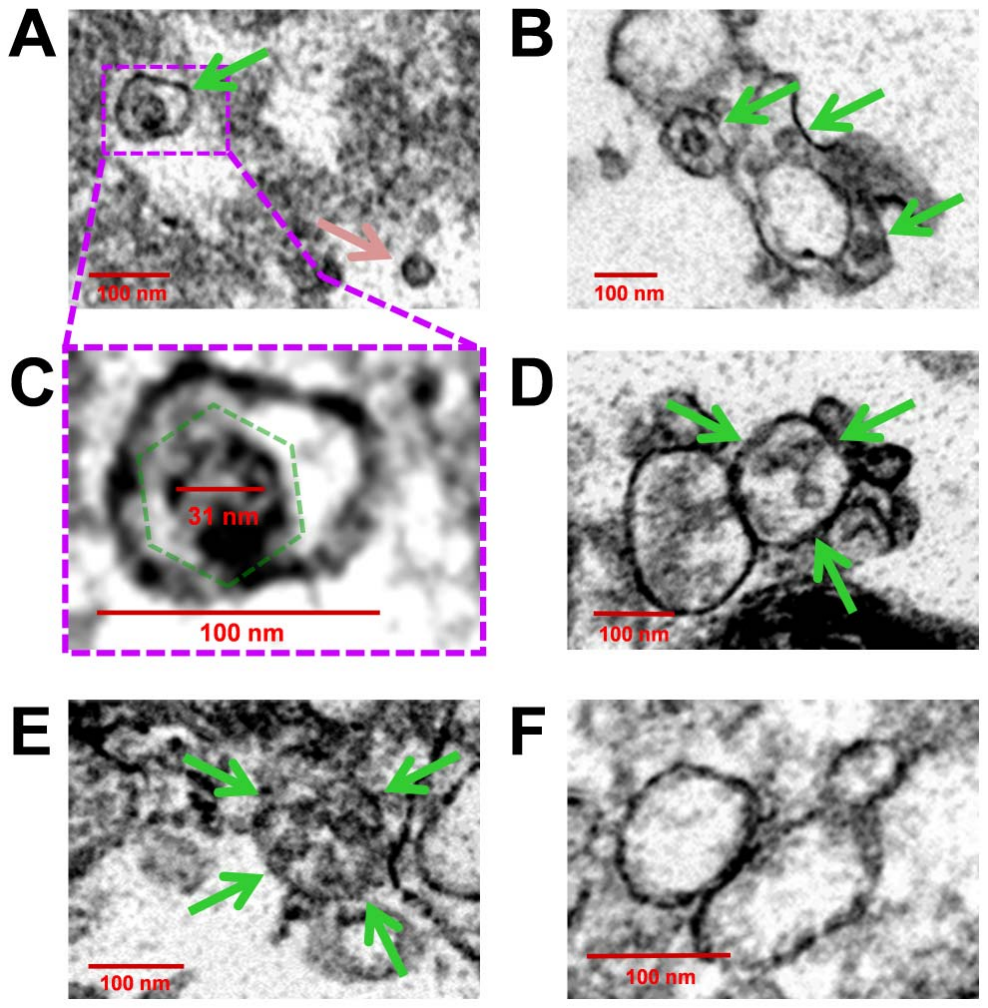
Virions were identified inside purified EMVs by transmission electron microscopy. EMVs were purified from mock-infected or infected differentiated C2C12 cells. Transmission electron microscopy was utilized to inspect EMVs for the presence of CVB3 virions. *(A)* Numerous virus-like particles within microvesicles (green arrow), although free virions was also observed (pink arrow). *(B)* EMVs harboring virus-like particles (green arrows) were of varying size. *(C)* Higher digital magnification (dashed purple box) of a virus-like particle revealed an icosahedral shape structure (dashed green polygon) slightly larger than 31 nm in diameter enclosed within a membrane structure. *(D)*, *(E)* Larger microvesicles also contained multiple virions (green arrows). *(F)* In contrast, microvesicles isolated from mock-infected C2C12 cells were identified by EM without virus-like particles.

We inspected the molecular structure of EMVs In order to clarify their association with infectious virions. Purified EMVs from Timer-CVB3 or mock-infected differentiated C2C12 cells were processed for transmission electron microscopy (TEM). For infected C2C212 cells, virus-like particles enclosed in membrane structures were readily observed by TEM (**Figure 13**). Single virions were seen within small microvesicles (**Figure 13A**; green arrow), although free virions were also observed in sections (**Figure 13A**; pink arrow). We suggest that free virions may represent EMV-associated virions which were disrupted during extensive processing for TEM. Also, virus-like particles were observed in EMVs of various sizes (**Figure 13B**; green arrows), perhaps reflecting the broad range of densities identified for infectious EMVs following iodixanol gradient purification. Higher digital magnification revealed the icosahedral shape of these virus-like structures (**Figure 13C**; dashed green polygon), and the diameter of virions was close to 31 nm - similar to previous studies describing the crystal structure of CV [44]. In some cases, EMVs contained three or four virions (**Figure 13D** and **Figure 13E**, respectively; green arrows). In contrast, microvesicles isolated from mock-infected C2C12 cells failed to harbor virus-like structures (**Figure 13F**). These microvesicles were more uniform in size (approximately 100–150 nm) and may represent traditional exosomes released by C2C12 cells [45].

## Discussion

Tracking viral infection may be critical in understanding viral dissemination and pathogenesis. For example, vaccinia virus has been shown to undergo repulsion of superinfection by the early expression of A33 and A36 protein which allows the virus to increase viral spread and maximize the replication rate substantially [46]. Also, human T-lymphotropic virus-1 (HTLV-1) utilizes the virological synapse, a specialized area of cell-cell contact promoting transmission of virus [47]. Much remains to be determined with regards to cell-to-cell spread of CVB3 and virus dissemination within the host [27]. The pancreas is a primary target organ for CVB3 infection in mice, and early viral replication here may seed other organs such as the heart and CNS. For example, transgenic mice expressing the antiviral molecule interferon-γ within the pancreas showed reduced viral titers and substantially less myocarditis [22]. Mice are considered an informative model to evaluate the mechanisms of coxsackievirus pathogenesis, and many of diseases caused by coxsackieviruses have been recapitulated in a mouse model [48]. Importantly, understanding factors contributing to early pancreatic infection may help to reduce viral replication and virus dissemination within the host. Therefore, we engineered the “fluorescent timer” protein into our infectious clone of CVB3 in order to monitor virus dissemination both in culture and *in vivo*. “Fluorescent timer” protein containing a nuclear localization signal has been previously utilized to track the cytoplasmic accumulation of nuclear proteins within infected cells [49]. However, this unique molecular marker has not been employed to follow the progression of virus infection directly.

By tracking the progression of virus infection utilizing Timer-CVB3, we observed intriguing aspects of virus replication in partially differentiated progenitor cells which we did not anticipate. First, we identified extensive intracellular membrane remodeling in partially differentiated NPSCs which reflecting the formation of viral replication organelles described by others [34]. By time-lapse video, the conversion of “fluorescent timer” protein from green to red as monitored by fluorescence microscopy revealed distinct regional viral replication organelles fluorescing in an asynchronous fashion. Previous studies have described the ability of viral 2B and 3A proteins of CVB3 to ablate protein trafficking and secretion by disrupting the Golgi apparatus [50] [51] [52]. Also, CVB3 has been shown to modulate cell host factors such as PI4KIIIβ, GBF1 and ARF1 in order to remodel intracellular membranes for efficient viral replication [53] [34] [54]. We suggest that ongoing translation during virus replication and the lack of virus protein transit due to compartmentalization and formation of CVB3 replication organelles within the host cell reflects the localized conversion of “fluorescent timer” protein from green to red as shown by time-lapse video.

The “fluorescence timer” protein contains a polyglycine linker and a 3C^pro^/3CD^pro^ cleavage site immediately downstream of the viral polyprotein start codon. Hence, “fluorescence timer” protein served as a marker for infected cells similar to other fluorescence proteins, such as eGFP and dsRED, described by our previous studies [26] [27]. Nevertheless, “fluorescence timer” protein may eventually diffuse away from sites of ongoing viral RNA translation and replication within the cell. Therefore, we determined colocalization of mature and recent viral “fluorescence timer” protein with viral 3A protein, an authentic viral protein previously shown to be closely associated with coxsackievirus replication complexes. Colocalization of “fluorescent timer” protein and viral 3A protein was readily observed in partially differentiated NPSCs infected with Timer-CVB3. Viral 3A protein has been shown previously to play a critical role in the reorganization of the secretory pathway and the generation of CVB3 replication organelles [34]. Therefore, following “fluorescent timer” protein signal during live imaging of infected cells may reveal the dynamics of intracellular membrane rearrangements critical for viral replication.

Based on these new data, we suggest that viral replication organelles physically retain proteins following viral RNA translation, and that potential diffusion of “fluorescence timer” protein away from these replication complexes may be restricted following the assembly of virus-modified intracellular membranes. Subsequently, the unique features of Timer-CVB3 have enabled the direct visualization of intracellular membrane remodeling of the infected host cell in real time. The induction and visualization of Timer-CVB3 replication organelles utilizing “fluorescent timer” protein also reflects the ability of enteroviruses to hijack the autophagy pathway and maximize viral replication [31] [30] [37] [55].

Utilizing Timer-CVB3, we monitored the generation of extracellular microvesicles (EMVs) containing viral material which may represent a novel strategy of virus shedding and transmission to neighboring cells. A large numbers of EMVs were released from partially differentiated NPSCs and in C2C12 myoblast stem cells following infection with Timer-CVB3. The extensive intracellular membrane remodeling observed in differentiated NPSCs and C2C12 cells following Timer-CVB3 infection may eventually contribute to microvesicle shedding similar to the formation of exosomes in cell lines, tumor cells and other cell lineages [45]. We initially considered the possibility that shed EMVs represented “cellular debris” released from dying cells. However, we feel that shed EMVs represent an ordered process commandeered by the virus to escape the host cell for at least five reasons: 1) First, we have previously shown little to no cytopathic effects in five and sixteen-day differentiated NPSCs infected with eGFP-CVB3 [12]. 2) Time-lapse photography revealed continuous shedding of intact EMVs from infected cells which remained alive and undamaged (**Figure 7**). 3) Shed EMVs characteristically contained the mature form of “fluorescence timer” protein, as opposed to recent, or recent plus mature “fluorescence protein” which might be expected if virus-induced cytopathology contributed to the unregulated release of cellular debris (**Figure 7**). 4) EMVs retained considerable stability in culture (**Figure 10**) and during substantial and lengthy procedures which included isopycnic gradient centrifugation and Exoquick-TC precipitation (**Figure 11**). 5) Finally, inspection of EMV-associated proteins (including LC3-II) and infectious virus indicated unique cellular protein signature patterns for EMVs, and the preferential association of virions with shed EMVs (**Figure 12**). We suggest that the release of EMVs harboring infectious virus represents an active and controlled process involving the autophagy pathway.

We examined the presence of autophagic proteins within EMVs released from differentiated NPSCs following CVB3 infection. Differentiated cells were transduced with LC3-GFP and infected with dsRED-CVB3. Shed microvesicles expressing both LC3-GFP and virus protein were readily observed. EMVs isolated from infected NPSCs and C2C12 cells were found to comprise high levels of infectious virus, and virus-like particles were readily observed in EMVs utilizing transmission electron microscopy (TEM). A broader range of densities in membrane-associated fractions were identified for infectious EMVs utilizing iodixanol gradient purification, as compared to enveloped hepatitis A viruses (eHAVs) resembling exosomes [41] [56]. These results are consistent with the broad size distribution range observed for EMVs (**Figure 11B**), and TEM data showing virion-comprising EMVs of various shapes and complex membrane morphology (**Figure 13**). Furthermore, western analysis of purified EMVs indicated the presence of viral protein 1 (VP1), flotillin-1 (a protein previous shown to be found in exosomes) and the preferential detection of the lipidated form of LC3 (LC3 II). These results suggest that the autophagic pathway contributes to CVB3 shedding similar to the previously described autophagosome-mediated exit without lysis (AWOL) model for poliovirus release [35]. Although proposed single-membrane vesicles derived from the autophagosome pathway and containing poliovirus have been suggested to be short-lived, our results with CVB3 suggest that these structures may be considerably more stable than anticipated [57]. Also, we recently described a reduction in the level of intracellular autophagosomes in differentiated NPSCs following CVB3 infection [28], an observation which contrasts with our results observed for HL-1 cells (cardiomyocyte cell line) or undifferentiated NPSCs infected with CVB3. Based on our new data, we speculate that the observed reduction of intracellular autophagosomes in differentiated NPSCs infected with CVB3 in our previous study is the result of the ejection of autophagosomes specifically within progenitor cells undergoing differentiation [43].

**Figure 14:**
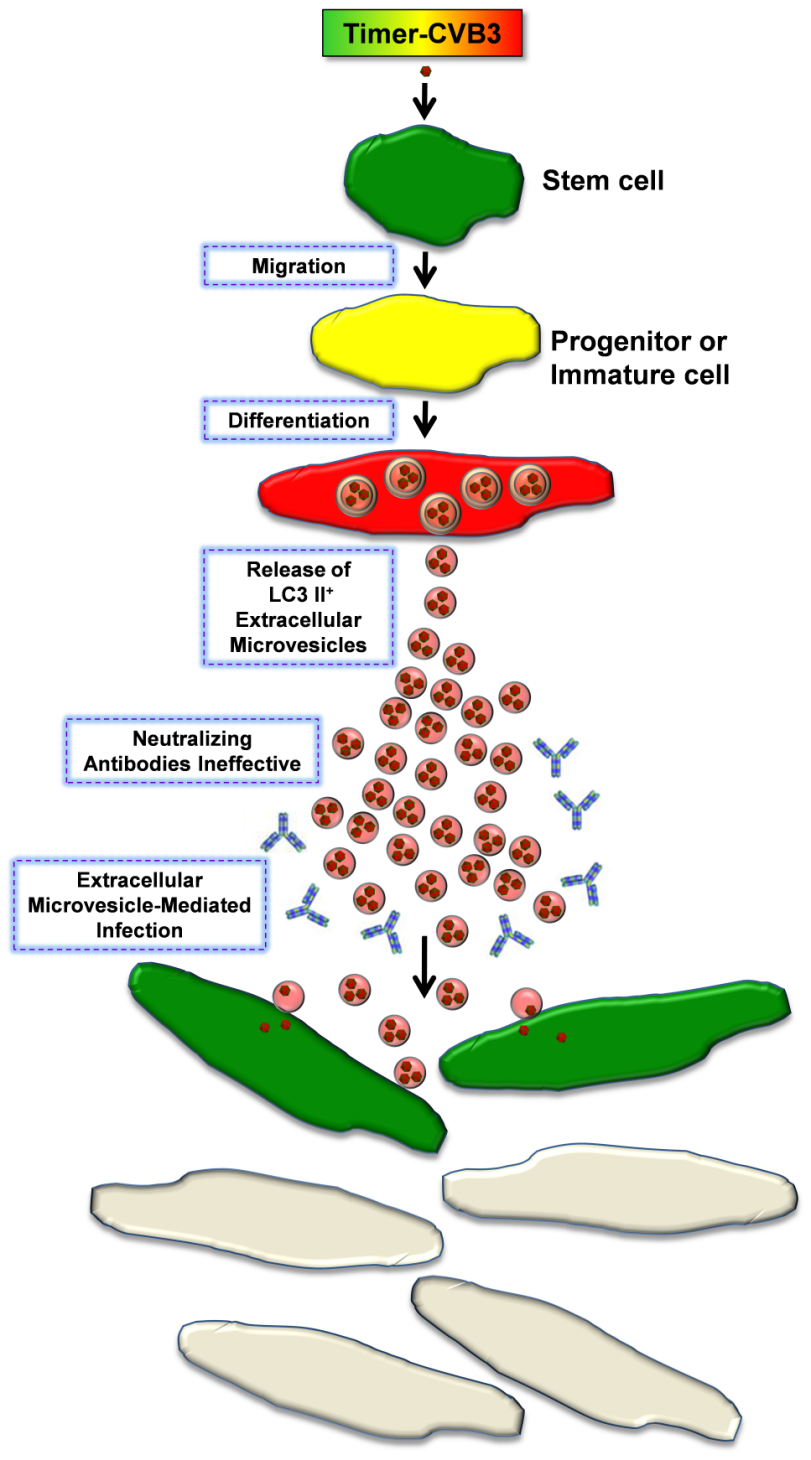
Model of Timer-CVB3 dissemination by EMVs triggered following stem cell migration and differentiation. NPSCs are highly susceptible to CVB3 infection. Upon progenitor cell migration and differentiation, LC3 II^+^ EMVs containing infectious virus are shed by cells. Both the differentiation process and viral infection enhance shedding of single membrane EMVs derived from the autophagy pathway. Neutralizing antibodies may be ineffective against infectious virus within the protected environment of the extracellular microvesicle. Fusion of EMVs with cells assists in CVB3 dissemination and expansion to new target cells, some of which do not express the CVB3 receptor for entry (CAR). New target cells are identified by the expression of recent viral protein (green).

Previous publications have suggested the possible contribution of EMVs or exosomes to virus dissemination [42] [58]. A recent study has shown that hepatitis C viral RNA transfer via exosomes are sufficient to activate plasmacytoid dendritic cells and induce an innate immune response in the host [59]. Also, hepatitis A virus, previously considered to be a non-enveloped virus, was recently revealed to hijack cellular membranes and escape the host cell as enveloped viruses resembling exosomes [41] [56]. Shed microvesicles share assembly pathways with retrotransposon elements and viruses, suggesting that viruses exploit cellular microvesicle pathways to maximize dissemination [60]. Also, double-membrane autophagic vesicles induced following enterovirus infection may fuse with the cell membrane leading to possible extracellular release of either single-membrane-enclosed or free virions from the cytosolic lumen [61]. Shed microvesicles produced in cells may ultimately assist in CVB3 egress from infected cells in the absence of cytolysis [35], and may provide a successful route of transmission to uninfected cells despite the presence of host neutralizing antibodies [62]. Our model of virus dissemination during the differentiation of progenitor cells by the production of EMVs, and the potential use of Timer-CVB3 to characterize this model is shown in **Figure 14**.

Although we have shown here that EMVs carry infectious virions, viral material shuttled by shed microvesicles during later stages of infection may be limited to infectious viral RNA – perhaps representing a novel strategy adapted by CVB3 to infect neighboring cells during persistence in the absence of virus capsid formation or cellular cytopathicity [20]. Supporting this idea, other groups have shown the ability of exosomes to transport RNAs or micro RNAs from cell to cell [42] [63]. The migratory nature of progenitor cells provides CVB3 with a unique vehicle to disseminate within the host [19], and that cellular differentiation may be a signal for the virus to escape by extracellular microvesicle-mediated discharge. Does CVB3 induce novel autophagosome ejection from infected cells, or does the virus commandeer a natural process for its own selfish purposes? We suggest that CVB3 may upregulate and exploit a natural process which may occur to a limited degree within progenitor cells during differentiation. EMV release may be significantly enhanced in differentiating cells either due to their inherent role in the differentiation process [64] [65] [66], or due to virus-induced enhancement and release during cellular differentiation. Also, we suggest that virus dissemination by EMVs may expand tropism through fusion with cells which fail to express the coxsackie and adenovirus receptor.

Our observation of red fluorescing particles nearby green fluorescing cells (shown in (**Figure 7J**, red arrow) provided the early inspiration for examining the presence of infectious shed microvesicles in Timer-CVB3-infected C2C12 cells. The unique qualities of Timer-CVB3 contributed to the formulation of our original hypothesis of shed infectious microvesicles by providing kinetic data from a static fluorescence image which would not normally be revealing. For example, if we had simply determined viral protein expression utilizing a single channel color for **Figure 7J**, we might have simply concluded the presence of cellular debris near an infected cell. Furthermore, the use of Timer-CVB3 in evaluating antiviral compounds of unknown function may provide additional mechanistic information for mode of action for a given compound. For example, an antiviral compound which may hypothetically act to inhibit virus release, although not initial entry, may lead to the presence synchronized green cells, followed later by red cells and the absence of newly-infected green cells. In contrast, an antiviral compound which decreases viral replication or translation may be reflected by the early appearance of dim green cells, followed later by both dim green and dim red-infected cells.

A possible limitation of observing “fluorescent timer” protein expression in infected tissue is that cells are required in this model to be observed directly, and some methodologies (such as flow cytometry or histology) necessitate significant processing time. However, the results of flow cytometry data using HeLa RW cells infected with Timer-CVB3 at various time points over 72 hours PI and fixed with 4% paraformaldehyde (**Figure 2**) demonstrate that the conversion of “fluorescent timer” protein can be stopped for subsequent analysis at a later time point. Timer-CVB3 will be of particular value when tracking infection *in vivo*, whereby initial time of infection is not clear, or in mixed cell cultures where one cell type may be first infected followed sequentially by less susceptible cell types. Timer-CVB3 will also assist in characterizing persistent infection in the heart or CNS, whereby the nature of persisting viral material is unclear. For example, new viral protein expression during sporadic infection or virus reactivation might be expected to produce green fluorescence in cells harboring Timer-CVB3 infection, while a carrier-state infection would produce red and green fluorescence simultaneously.

Future studies will focus on isolating infectious EMVs from the sera of mice in the presence of an ongoing neutralizing antibody response. Also, we will determine the ability of shed microvesicles to expand the tropism of CVB3, and identify inhibitors of microvesicle fusion which might act as novel antiviral compounds.

## Materials and Methods

### Ethics statement

This study was carried out in strict accordance with the requirements pertaining to animal subjects protections within the Public Health Service Policy and USDA Animal Welfare Regulations. All experimental procedures with mice were approved by the San Diego State University Institutional Animal Care and Use Committee (Animal Protocol Form #10-05-013F), and all efforts were made to minimize suffering.

### Generation of Timer-CVB3 stock

The Timer gene was amplified from the pTimer plasmid (Clontech Inc.) with primers containing *Sfi*I restriction site sequences. Following amplification, the PCR product was cut with Sfi1 and cloned into our parental CVB3 vector (pMKS1) linearized with *Sfi*I [25]. Also, a CMV promoter was cloned into the Timer-CVB3 plasmid. The plasmid was precipitated by ethanol incubation at −20°C for 12 hours. After centrifugation and washing in 70% ethanol, the DNA pellet was air-dried at room temperature for 20 minutes. The pellet was then resuspended in sterile, nano-filtered water at a concentration of 1 microgram/microliter.

The generation of recombinant CVB3s has been described previously [25] [26]. Timer-CVB3 was produced as follows: HeLa cells were transfected with 2.8 microgram of the sterile Timer-CVB3 plasmid using Lipofectamine 2000 (Invitrogen) and incubated at 37°C, 5% CO_2_ for 3 hours. The transfection solution was removed and the wells were washed in 3 mL of 1×DMEM with 10% FBS. Wash was removed and 2 mL of 1× DMEM with 10% FBS was added. The transfected cells were incubated at 37°C, 5% CO_2_ for 1day. After 1 day incubation, an additional 2 mL of 1× DMEM with 10% FBS was added to each well of transfected cells on the plate. The plate was incubated at 37°C, 5% CO_2_ for 2 days.

Transfected cells were observed under fluorescence microscope until the cells were observed to first fluoresce green followed by fluorescing red. Supernatants were transferred to a 50 mL conical, screw-cap tube, and 2 mL of 1× DMEM was placed on top of the cells. The 50 mL screw-cap tube was centrifuged at 2000 RPM for 2 minutes. The supernatant was transferred to a new 50 mL conical screw-cap tube and cells were removed by cell-scraping. The cell solution was transferred to the pellet in the first 50 mL conical, screw-cap tube, and the pellet was resuspended in the solution. The cell solution was then lysed by freeze thaw 3 times: 5 minutes of freezing in 95% ethanol with dry-ice, 30 minutes in ice water, and 5 minutes at room temperature with rocking. The freeze-thawed solution was then centrifuged at 2000 RPM for 2 minutes. The supernatant from this tube was transferred to the original supernatant from the first centrifugation. This solution was then vacuum-filtered through a filter with 0.2 µm pore. This virus solution was labeled passage 1 and used to infect HeLa cells in a 150 mL flask.

HeLa cells grown to 80% confluency were washed two times in 10 mL of 1× PBS. Then, 8 mL of Timer-CVB3 (Passage 1) was added to HeLa cells and incubated at 37°C, 5% CO_2_ for 1 hour, and the flask was rocked every 15 minutes to fully cover the cells. Then, 18 mL of 1× DMEM with 10% FBS was added and the flask was incubated at 37°C, 5% CO_2_ for an additional 48 hours. Passage 2 supernatant and cells were separated, and cells were scraped into 10 mL of 1× DMEM, lysed by freeze thaw as described for Passage 1. This virus solution was titrated by plaque assay using HeLa cells at 37°C, 5% CO_2_ for 48 hours and stored at −80°C.

### Plaque assay

Virus titrations were carried out as described previously [26]. Briefly, HeLa cells were diluted to a concentration of 1×10^5^ cells/mL, and 3 mL of the cell solution was added to each well in 6-well plates. The plate was incubated at 37°C, 5% CO_2_ overnight. The next day, serial dilutions were made for each viral sample. After the 1 hour infection, each well was filled with 4 mL of 0.6% Agar, 1× DMEM + 2.5% FBS and 1× Penicillin/Streptomycin (P/S). The plate was incubated at 37°C, 5% CO_2_ for approximately 44 hrs before fixing. After fixing, 1 mL of 0.25% crystal violet was added to each well, and the plate was incubated at room temperature for 1 hour. After staining, plaques were counted.

### Flow cytometry

HeLa RW cultures were added to 6-well plates as described above. After ∼24 hours, cells were mock-infected, infected with eGFP-CVB3 or dsRED-CVB3 at an MOI of 0.1. Alternatively, cells were infected with Timer-CVB3 at an MOI of 0.01 or 0.1. After 12, 24, 36, 48, or 72 hours PI, cells were fixed in 4% para-formaldehyde and washed three times in 1× PBS. Cells were stored in 1× PBS with 1% BSA at 4°C. Cells were analyzed using a BD FACSAria located in the SDSU Flow Cytometry Core Facility.

### Isolation, culture, and infection of neurospheres

Neurospheres were isolated and cultured as previously described [12]. Neurospheres were vigorously dissociated and resuspended in complete NPSC culture medium to a concentration of 10^5^ cells/mL in a 24 well plate and infected with Timer-CVB3 at an MOI of 0.1. Cultures were inspected daily using a Zeiss Axio Observer D.1 inverted fluorescent microscope. NPSCs infected with Timer-CVB3 could establish carrier-state infection, as described previously for eGFP-CVB3 [12]. These carrier-state infections were inspected for “fluorescent timer” protein at the indicated time. Parallel infections were also performed on NPSCs continuously grown and passaged in NPSCs media containing 10 ug/mL Poly IC. Alternatively, 10^5^ cells/ml of NPSCs were differentiated in a 4-well chamber slide (1 ml per well) as previously described [12] and in a u-dish (iBidi, Inc., cat#81156). After differentiation for 5 days, differentiated NPSCs were infected with Timer-CVB3 at an MOI of 0.1. Following infection, differentiated cells in the u-dish were monitored daily for the expression of Timer protein. Once the expression of “fluorescent timer” protein was observed, the culture was imaged at 10 minute intervals, holding the field of image, magnification, channel exposure times, and focus consistent. The focus was adjusted occasionally as needed.

### Infection of differentiated NPSCs

NPSCs were plated onto gelatin/fibronectin-coated micro dishes containing differentiation media at a cell concentration of 10^5^ cells/ml as described previously [12] [28]. The cells were differentiated for 5 days and then infected with Timer-CVB3 (moi = 0.1). Alternatively, differentiated NPSCs were infected with dsRed-CVB3 (moi of 0.1) and transduced with a recombinant adenovirus expressing GFP-LC3 (Adeno-GFP-LC3). Following infection, the cells were observed by fluorescent microscopy at magnification of 320× at the indicated time points.

### Immunofluorescence microscopy

At 3 or 4 days PI, differentiated cultures in the chamber slides were fixed in 4% para-formaldehyde and washed three times in PBS. Fixed cells were permeabilized with 0.25% TritonX-100 in PBS, washed three times, blocked with 10% normal goat serum (NGS) and immunostained using the following antibodies: Nestin (Covance Inc.; Cat# PRB-315C) at 1∶1000, neuronal class III β-tubulin (Covance Inc.; Cat# PRB-435P) at 1∶1000, GFAP (Sigma-Aldrich Co.; Cat# G 9269) at 1∶1000, MBP (Chemicon Inc.; Cat# AB980) at 1∶1000, Viral 3A protein [51] at 1∶100. Primary antibodies were diluted in 2% Normal Goat Serum (NGS) in PBS (150–200 µl per slide) in humidified chamber and incubated overnight. Slides were washed with PBS for 5 min (3×). Secondary antibodies (at 1∶1000) conjugated to Alexa-Fluor-647 (Invitrogen; Cat# A21245), were diluted with 2% NGS in PBS (150–200 µl per slide) and incubated overnight. Following incubation with the secondary antibody, slides were washed with PBS for 5 min (3×), and mounted in Vectashield with DAPI. Three to five representative images of the cultures were taken for each sampling time point at multiple magnifications.

### Infection, imaging, and image analysis of HeLa RW cell cultures

HeLa RW cultures were plated in a 6-well plate at a concentration of 10^5^ cells/ml (3 ml per well), treated with either 10 µg/ml Poly IC, 50 µ/ml IFN-β, or media alone and allowed to adhere to the plate overnight. Timer-CVB3 was then added at moi = 0.01 (14 µl of 10^6^ pfu/mL virus stock) or moi = 0.1 (14 µl of 106 pfu/mL virus stock). Eight images of each culture were taken at 100× total magnification per condition, per day. Samples for viral titrations were taken immediately following imaging. For each image, the number of red, yellow, green, or round colorless cells were counted using the ImageJ Cell Counter plug-in.

### Isopycnic gradient centrifugation of EMVs

EMVs were purified from differentiated C2C12 cells. C2C12 cells were seeded onto T-25 flasks in DMEM supplemented with 10% fetal bovine serum + antibiotics and infected with eGFP-CVB3 (moi = 100; 2.9×10^8^ pfu/mL virus stock concentration). A gradient maker was used establish a continuous 8–20% iodixanol gradient (Opti-Prep; Sigma-Aldrich Co.). 1 ml of day 5 PI supernatant isolated from infected C2C12 cells was used for the continuous 8–20% iodixanol gradient, and the gradient was centrifuged at 141,000 g (28,700 rpm) in an SW.41 Ti rotor for 48 h at 4C in a Beckman L8-60 Ultracentrifuge. Twenty-four fractions were collected from the top of the gradient and the density for each fraction was determined using a Bausch & Lomb refractometer (Bausch & Lomb, Inc.). Fractions were frozen at −70°C prior to virus titration by plaque assay.

### EMV isolation by Exoquick-TC and western blots

EMVs were purified from differentiated C2C12 cells and NPSCs. C2C12 cells were seeded onto 6-well culture plates at a concentration of 10^5^ cells/well in DMEM supplemented with 10% fetal bovine serum + antibiotics and infected with eGFP-CVB3 (moi = 100; 14 µl of 10^9^ pfu/mL virus stock). NPSCs were plated onto gelatin/fibronectin-coated micro dishes at a cell concentration of 10^5^ cells/ml. NPSCs were differentiated for 5 days, and media was changed every two days. NPSCs were infected with eGFP-CVB3 (moi = 0.1; 14 µl of 10^6^ pfu/mL virus stock). On day 3 PI, supernatants from T-75 flasks were isolated and centrifuged at 3000 g (rcf) for 15 min to spin down cell debris and resuspended in 1× DMEM. Supernatants were transferred to a new conical tube, and 2 ml Exoquick-TC (System Biosciences Inc) was added for every 10 ml of supernatant. The mixture was incubated overnight at 4°C, and EMVs were centrifuged at 1500 g (rcf) for 30 min. Purified EMVs were resuspended in 100 µl 1× PBS. 20 µl of this mixture was placed on a slide and visualized by fluorescence microscopy at 320× magnification. Also, HeLa cells were infected with 50 µl of the purified EMVs and observed by fluorescence microscopy at various time points post-incubation. EMV and non-EMV fractions were freeze/thawed three times, and viral titers were determined by standard plaque. Alternatively, scraped cells or purified EMVs were washed with phosphate buffered saline and then disrupted with chilled RIPA buffer, and incubated on ice for 30 min with vortexing every 10 min. Protein concentrations were measured by bicinchoninic acid assay. Three primary antibodies were used for western blotting: rabbit anti-LC3 A/B (Cell Signaling Technologies, Inc, Cat# 4108), rabbit anti-Flotillin-1 (Cell Signaling Technologies, Inc, Cat# 3253), and mouse anti-enteroviral VP1 (Vector Laboratories Cat# VP-E603). 20 µg of protein derived from purified EMVs or cell lysates were utilized for western blot analysis, as described previously [28].

### Transmission electron microscopy

EMVs were purified from differentiated C2C12 mock-infected or infected with Timer-CVB3. Purified EMVs were resuspended in 1 ml 2.5% glutaraldehyde/PBS and incubated on ice for 1.5 hours. Samples were centrifuged at 4000× g for 20 minutes at 4°C. Pellets were washed without resuspension twice with PBS for 10 minutes. Pellets were then resuspended in 250 µl PBS. The resuspended pellets were centrifuged at 4000× g for 20 minutes at 4°C. Supernatants were removed and pellets were resuspended in 1 ml 1% osmium tetroxide in PBS. The resuspended pellets were incubated on ice in the dark for 1 hour. Samples were centrifuged and washed as previously described. Following the removal of 1% osmium tetroxide, 1% uranyl acetate in PBS was added to the pellets without resuspension and incubated on ice in the dark for 1 hour. Pellets were washed with distilled water three times without resuspension for 10 minutes. Following the removal of distilled water, pellets were then dehydrated with a step-wise ethanol gradient (30%, 50%, 70%, 85%, 95%) for 10 minutes per step without resuspension. Pellets were then washed with 100% acetone three times for 10 minutes per wash. Following acetone removal, 33% EPON/66% acetone solution was then added to pellets without resuspension and placed in spin wheel overnight. Following removal of 33% EPON/66% acetone, 66% EPON/33% acetone solution was added to pellets without resuspension and placed in spin wheel overnight. Following 66% EPON/33% acetone removal, 100% EPON was added to pellet (without resuspension) and incubated at 60°C overnight to harden. Samples were cut into <50 nm sections using a diamond knife and a Leica EM UC6 microtome. Sections were positively stained using lead citrate [67] and imaged using a FEI Tecnai 12 transmission electron microscope.

## Supporting Information

Figure S1 **Progression of Timer-CVB3 infection in HeLa cells treated with ribavirin.** HeLa cells were infected with Timer-CVB3 (moi = 0.01 or 0.1) in the presence or absence of ribavirin at 10 or 100 µg/mL. *(A)* At low moi (moi = 0.01), 100 µg/mL ribavirin treatment greatly reduced “fluorescent timer” protein expression in infected HeLa cells at 32 and 48 hours PI. Also, fewer signs of cytopathic effects were observed in ribavirin-treated cells at 48 hours PI. *(B)* At the higher moi (moi = 0.1), 100 µg/mL ribavirin treatment also significantly reduced “fluorescent timer” protein expression in infected HeLa cells at 32 and 48 hours PI. Also, cytopathic effects were delayed by 16 hours in HeLa cells treated with 100 µg/mL ribavirin.
(TIF)

Figure S2 **Ribavirin treatment restricted the progression of Timer-CVB3 infection in HeLa cells.** HeLa cells were infected with Timer-CVB3 (moi = 0.01 or 0.1) in the presence or absence of ribavirin at 10 or 100 µg/mL. *(A)* At low moi, HeLa cells treated with 100 µg/mL ribavirin showed fewer signs of cytopathic effects (round colorless cells – grey bars) and fewer green, yellow, or red cells by fluorescence microscopy following infection with Timer-CVB3 as compared to untreated cultures at 32 and 48 hours PI. *(B)* At higher moi, Ribavirin treatment at 100 µg/mL also reduced the progression of “fluorescent timer” protein expression at 32 and 48 hours PI. Also, a delay in cytopathic effects was observed at early time points (24 and 32 hours PI). *(C)*, *(D)* A stepwise reduction in viral titers was observed in HeLa cells infected at a low moi and treated with ribavirin at 10 or 100 µg/mL. Also, viral titers were greatly reduced in HeLa cells infected at a higher moi and treated with ribavirin at 100 µg/mL.
(TIF)

Video S1 **Time-lapse video of differentiated NPSCs infected with Timer-CVB3.** “Fluorescent timer” protein changed from green to red over the span of 6 hours in differentiated NPSCs infected with Timer-CVB3 and observed by time-lapse video.
(MOV)

Video S2 **Time-lapse video of differentiated NPSCs infected with Timer-CVB3 at higher magnification – region 1.** “Fluorescent timer” protein changed from green to red over the span of 6 hours in differentiated NPSCs infected with Timer-CVB3 and observed by time-lapse video at higher magnification shown for region 1 (boxed region on the accompanying image).
(MOV)

Video S3 **Time-lapse video of differentiated NPSCs infected with Timer-CVB3 at higher magnification - region 2.** “Fluorescent timer” protein changed from green to red over the span of 6 hours in differentiated NPSCs infected with Timer-CVB3 and observed by time-lapse video at higher magnification shown for region 2 (boxed region on the accompanying image).
(MOV)

Video S4 **Time-lapse video of differentiated NPSCs infected with Timer-CVB3 at higher magnification - region 3.** “Fluorescent timer” protein changed from green to red over the span of 6 hours in differentiated NPSCs infected with Timer-CVB3 and observed by time-lapse video at higher magnification shown for region 3 (boxed region on the accompanying image).
(MOV)
