# Supplementary figures and images for: Coxsackievirus B Exits the Host Cell in Shed Microvesicles Displaying Autophagosomal Markers
Source: PLoS Pathog. 2014 Apr 10;10(4):e1004045. doi: 10.1371/journal.ppat.1004045 (PMC3983045; doi:10.1371/journal.ppat.1004045)

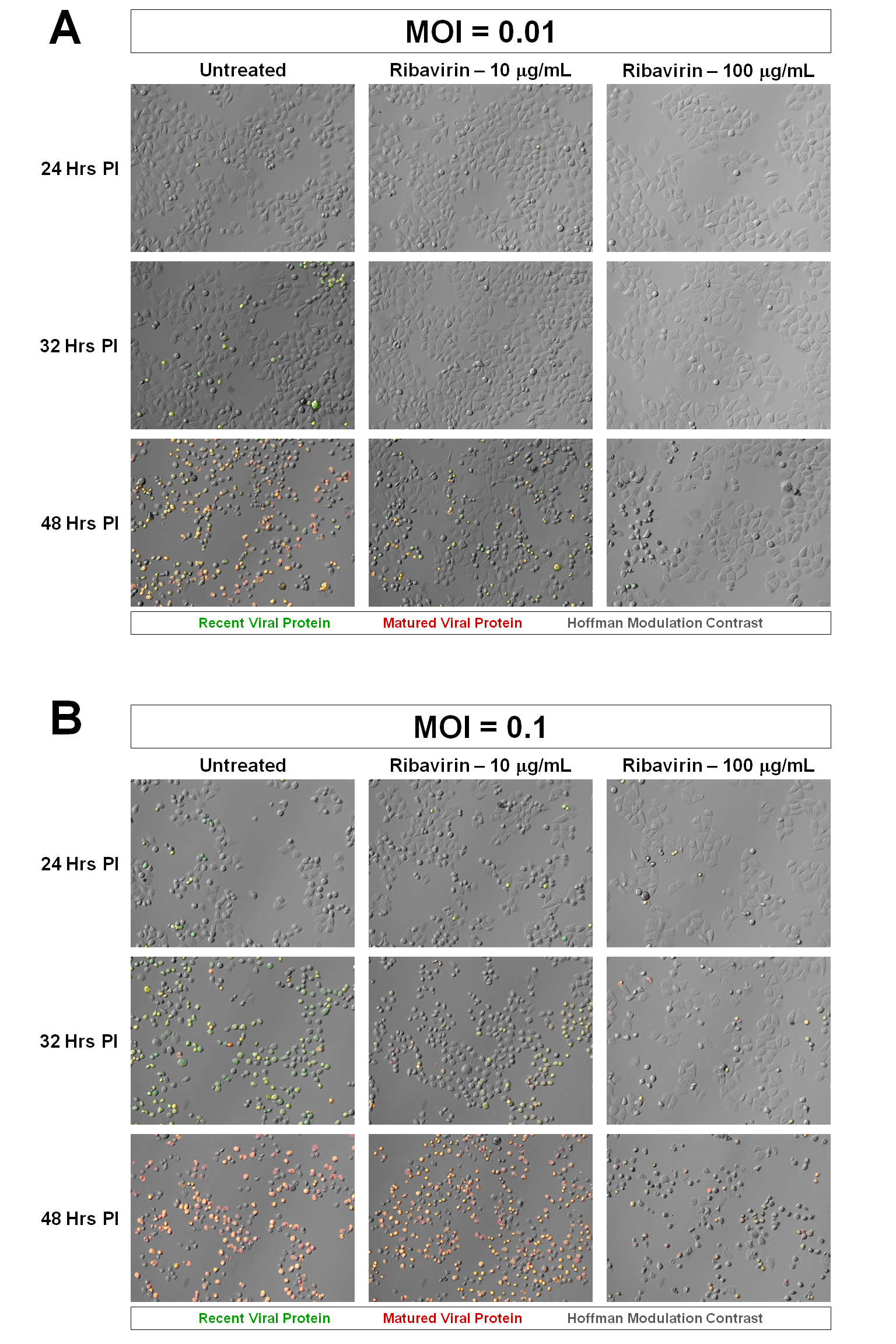

Supplement: Figure S1 — Progression of Timer-CVB3 infection in HeLa cells treated with ribavirin. HeLa cells were infected with Timer-CVB3 (moi = 0.01 or 0.1) in the presence or absence of ribavirin at 10 or 100 µg/mL. (A) At low moi (moi = 0.01), 100 µg/mL ribavirin treatment greatly reduced “fluorescent timer” protein expression in infected HeLa cells at 32 and 48 hours PI. Also, fewer signs of cytopathic effects were observed in ribavirin-treated cells at 48 hours PI. (B) At the higher moi (moi = 0.1), 100 µg/mL ribavirin treatment also significantly reduced “fluorescent timer” protein expression in infected HeLa cells at 32 and 48 hours PI. Also, cytopathic effects were delayed by 16 hours in HeLa cells treated with 100 µg/mL ribavirin. (TIF) [file ppat.1004045.s001.tif]

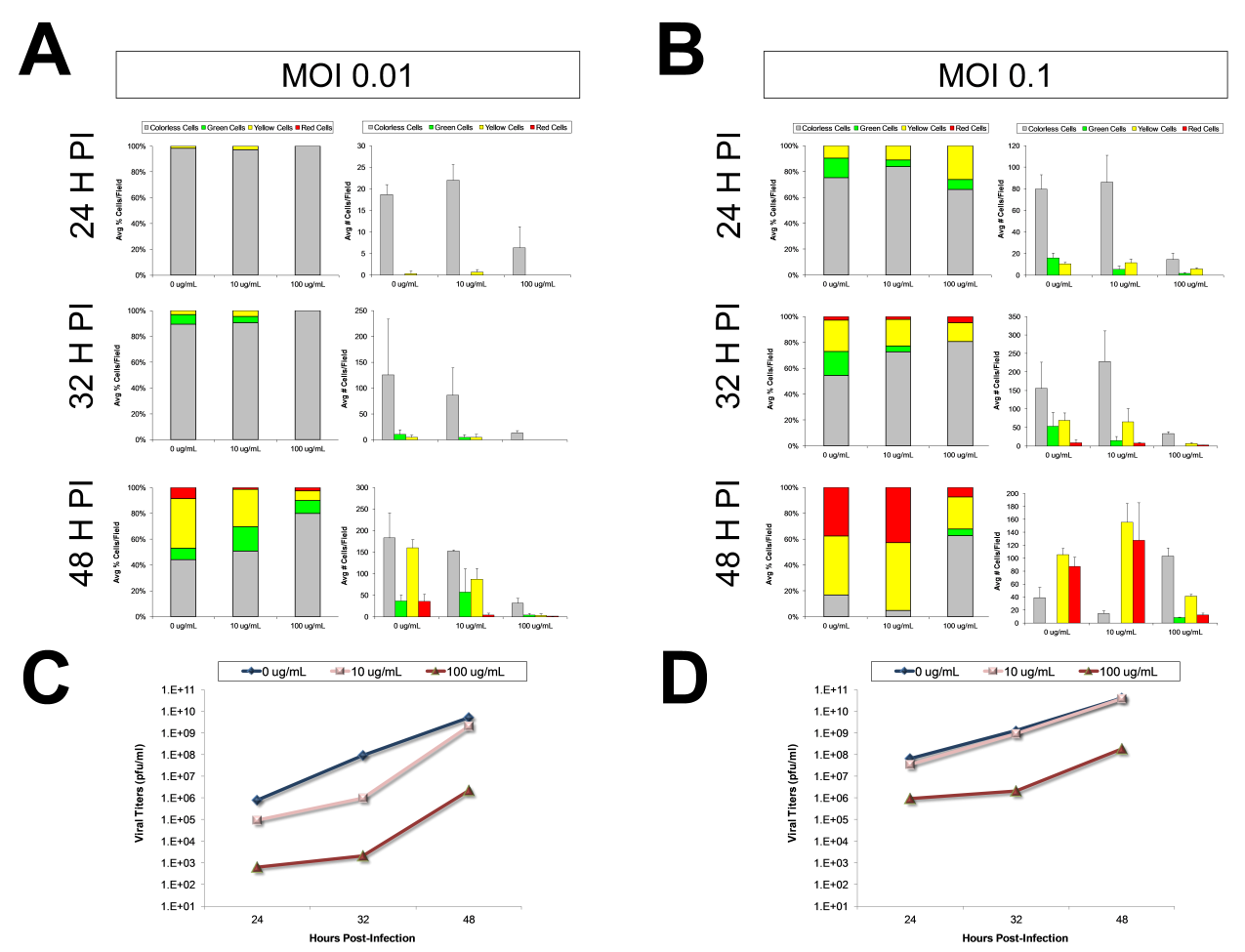

Supplement: Figure S2 — Ribavirin treatment restricted the progression of Timer-CVB3 infection in HeLa cells. HeLa cells were infected with Timer-CVB3 (moi = 0.01 or 0.1) in the presence or absence of ribavirin at 10 or 100 µg/mL. (A) At low moi, HeLa cells treated with 100 µg/mL ribavirin showed fewer signs of cytopathic effects (round colorless cells – grey bars) and fewer green, yellow, or red cells by fluorescence microscopy following infection with Timer-CVB3 as compared to untreated cultures at 32 and 48 hours PI. (B) At higher moi, Ribavirin treatment at 100 µg/mL also reduced the progression of “fluorescent timer” protein expression at 32 and 48 hours PI. Also, a delay in cytopathic effects was observed at early time points (24 and 32 hours PI). (C), (D) A stepwise reduction in viral titers was observed in HeLa cells infected at a low moi and treated with ribavirin at 10 or 100 µg/mL. Also, viral titers were greatly reduced in HeLa cells infected at a higher moi and treated with ribavirin at 100 µg/mL. (TIF) [file ppat.1004045.s002.tif]
